# Genome-wide analysis of the *Dof* gene family in durian reveals fruit ripening-associated and cultivar-dependent Dof transcription factors

**DOI:** 10.1038/s41598-019-48601-7

**Published:** 2019-08-20

**Authors:** Gholamreza Khaksar, Wassakarn Sangchay, Pinnapat Pinsorn, Lalida Sangpong, Supaart Sirikantaramas

**Affiliations:** 1 Molecular Crop Research Unit, Department of Biochemistry, Faculty of Science, Chulalongkorn University, 254 Phayathai Road, Bangkok, 10330 Thailand; 2 Omics Sciences and Bioinformatics Center, Chulalongkorn University, 254 Phayathai Road, Bangkok, 10330 Thailand

**Keywords:** Plant molecular biology, Natural variation in plants

## Abstract

DNA binding with one finger (Dof) proteins constitute a ubiquitous plant-specific transcription factor (TF) family associated with diverse biological processes, including ripening. We conducted a genome-wide analysis of durian (*Durio zibethinus* Murr.) and identified 24 durian *Dofs* (*DzDofs*), 15 of which were expressed in fruit pulp. Gene expression analysis revealed differential expression of *DzDofs* during ripening in two commercial durian cultivars from Thailand, Monthong and Chanee. Comparing the expression levels of fruit pulp-expressed *DzDofs* between cultivars revealed ten potential cultivar-dependent Dofs, among which *DzDof2*.*2* showed a significantly greater fold increase at every ripening stage in Chanee than in Monthong. The prediction of DzDof2.2’s function based on its orthologue in *Arabidopsis* revealed its possible role in regulating auxin biosynthesis. We observed significantly higher auxin levels during ripening of Chanee than Monthong which concurred with the greater expression of auxin biosynthetic genes. Transient expression of *DzDof2*.*2* in *Nicotiana benthamiana* significantly upregulated the expression levels of auxin biosynthetic genes. Higher expression levels of *DzDof2*.*2* in Chanee would enhance auxin levels through transcriptional regulation of auxin biosynthetic genes. Higher auxin levels in Chanee could activate auxin-mediated transcription, contributing to its faster ripening compared to Monthong through earlier initiation of the ethylene response (auxin-ethylene crosstalk).

## Introduction

Transcription factors (TFs) are pivotal regulators of the gene expression networks that control plant growth, development, and responses to environmental stimuli. The functional characterization of TFs can provide new insights into how plants grow and respond to numerous biotic and abiotic stressors. The DNA binding with one finger (Dof) proteins constitute a ubiquitous, plant-specific TF family. All Dof TFs harbour a highly conserved domain of about 50–52 amino acids (known as the Dof domain), which is always found at their N-terminus. The Dof domain has a Cys2/Cys2 Zn^2+^ finger structure that specifically binds to the cis-element of AAAG in the promoter of its targeted genes^1,2^. The C-terminuses of Dofs are highly variable, and act as transcriptional regulators of the genes responsive to them^3^. Dof TFs are associated with a wide range of biological processes, such as seed germination, light responses, flowering, carbon metabolism, plant hormonal signalling, and responses to both biotic and abiotic stressors^3–5^. The first isolation of a Dof TF was carried out in maize^6^. Since then, many studies have documented the Dof TFs in other plants, such as *Arabidopsis*^7^, tomato^5^, rice^8^, cucumber^9^, pepper^10^, banana^11^, and kiwifruit^12^. In a study by Skirycz *et al*.^13^, *Arabidopsis Dof1*.*1* (*AtDof1*.*1*, OBP2) was shown to regulate glucosinolate biosynthesis. Moreover, auxin levels in leaves and seedlings of OBP2 over-expressing lines were higher than those in wild-type plants. *AtDof5*.*4* (OBP4) was reported to be involved in transcriptional regulation of the cell cycle^14^. In addition, higher expression levels of OBP4 under exposure to abiotic stressors (salt and heat) and exogenous auxin were also documented^14^. However, our knowledge of the actual roles of Dof TFs during the ripening of climacteric fruit is relatively limited. Notably, Feng *et al*.^11^ identified four ethylene-inducible Dofs that were expressed at increasingly high levels during banana ripening. This was the first report of a possible role for Dof TFs in climacteric ripening. In a study by Zhang *et al*.^12^, the role of a kiwifruit Dof (AdDof3) in starch degradation during ripening was investigated. AdDof3 was shown to physically interact with the *AdBAM3L* (β-amylase) promoter, a key gene involved in starch degradation.

Durian (*Durio zibethinus* Murr.) is an economically important tropical fruit crop native to Southeast Asia, which has recently begun to be distributed globally. Durian is distinctive for its strong and unique odour, formidable spiny husk, and overwhelming flavour. It plays a vital role as an important export commodity in the Southeast Asian region. Thailand is the top exporter of durian across this region. There are more than 200 durian cultivars, but only a few cultivars are commercially grown and are in high demand in the international market. In Thailand, two cultivars, Monthong (*D*. *zibethinus* Murr. cv. ‘Monthong’) and Chanee (*D*. *zibethinus* Murr. cv. ‘Chanee’) are cultivated commercially in several provinces, particularly in the eastern and southern parts of Thailand^15^. Notably, these two cultivars exhibit distinctive sensory characteristics from one another. Monthong is a less sweet cultivar with a mild odour and light yellowish pulp, whereas Chanee has an overpowering aroma, sweeter taste, and dark yellowish pulp. Generally, Thai farmers harvest durian at the commercially mature stage, which occurs around 90 and 105 days after anthesis for Chanee and Monthong, respectively. Harvesting is done at the mature stage to facilitate fruit handling and transportation, protect the fruits from pathogen infection on the tree, and enhance their sensory characteristics. Chanee ripens four days after harvesting at the mature stage, whereas Monthong has a longer post-harvest ripening period that requires five days after harvesting at the mature stage. Given that durian is a climacteric fruit, its shelf life is restricted once the ripening process starts. This phenomenon could negatively affect the handling and transportation of durian and cause major economic losses. Therefore, gaining a deeper understanding of the ripening process in durian is of the utmost importance to control its post-harvest ripening. Despite the rising importance of durian as an economic fruit crop, genomic research on this species is almost non-existent, especially with regard to its Dof TFs. Our study aimed to conduct a genome-wide analysis to identify the Dof TFs in the durian genome, which has recently been published^16^. Further, we performed some routine *in silico* analyses, including gene structure analysis, identification of conserved motifs, and phylogenetic analysis. Finally, we analysed the expression patterns of durian Dofs (*DzDof*s) during post-harvest ripening in both the Monthong and Chanee cultivars and identified ripening-associated and cultivar-dependent *DzDof*s. The findings of our study deepen our knowledge of the role of Dof TFs during post-harvest ripening of climacteric durian fruits, and how differential expression patterns of some Dofs could contribute to differences in the ripening behaviours of the Monthong and Chanee cultivars.

## Results

### Identification of 24 DzDof transcription factors

**Figure 1:**
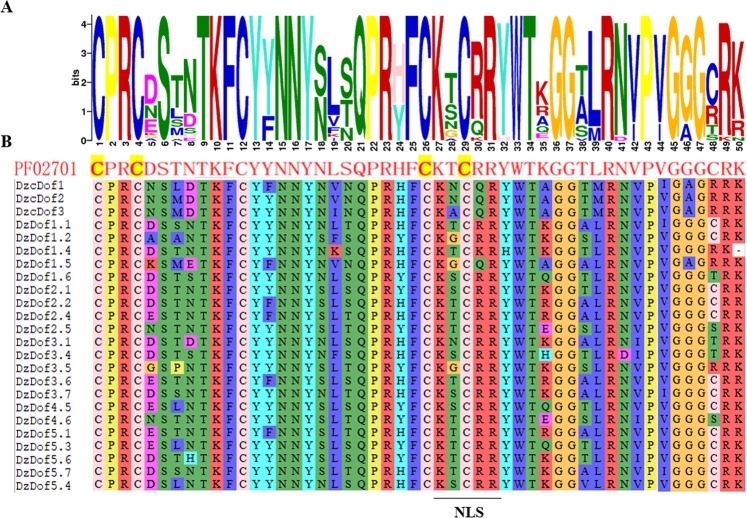
Multiple sequence alignment of the conserved Dof domains of 24 DzDofs. (**A**) Sequence representation LOGO obtained from multiple sequence alignment of the DzDof motifs. (**B**) Multiple sequence alignment of DzDof motifs. The nuclear localization signal (NLS) motif is also indicated in the alignment. PF02701 is the protein family ID of the zinc finger Dof domain retrieved from http://pfam.xfam.org/family/zf-Dof. DzcDof is durian cyclic Dof.

Our genome-wide attempts to identify *DzDofs* revealed a total of 24 genes encoding transcription factors of the DzDof family in Musang King genome, which was equal to the number of previously annotated *DzDofs* in the durian genome^16^ (Table 1). The sizes of the full-length coding sequences of *DzDof* genes ranged from 519 to 1560 bp. The deduced amino acid lengths of proteins made from *DzDofs* varied from 172 to 519 amino acids, with the isoelectric point (*p*I) of the resultant proteins predicted to range from 4.88 to 9.65 and their molecular masses from 19.19 to 56.59 kD (Table 1). Notably, DzDof2.4 and DzDof5.1 showed the highest levels of sequence similarity (89.85%), whereas DzDof1.2 and durian cyclic Dof1 (DzcDof1) were the least similar (22.82%) (Supplementary Table S1). Multiple sequence alignment of DzDofs revealed a highly conserved DNA binding domain of 50 amino acids at the N-terminus, structured as a Cys2/Cys2 Zn^2+^ finger (Fig. 1). This conserved motif, designated as the Dof domain, is the defining characteristic of Dof TFs. Moreover, a nuclear localization signal (NLS) was also detected in all DzDofs by *in silico* analyses. This sequence, which partly overlapped with the Dof domain, indicated the point of the nuclear localization of DzDofs.

**Table 1:** Dof transcription factors identified in durian (*Durio zibethinus* Murr.).

Gene name	Corresponding gene ID	ORF length (bp)	Protein length (aa)	MW (kD)	*p*I value
Durian cyclic Dof1 (DzcDof1)	XM_022890912.1	1443	480	52.01	6.37
Durian cyclic Dof2 (DzcDof2)	XM_022884508.1	1560	519	56.59	5.95
Durian cyclic Dof3 (DzcDof3)	XM_022882662.1	1458	485	53.17	6.77
DzDof1.1	XM_022885861.1	996	331	35.09	9.02
DzDof1.2	XM_022897927.1	927	308	34.52	4.88
DzDof1.4	XM_022864763.1	930	309	34.38	9.45
DzDof1.5	XM_022921573.1	519	172	19.19	9.15
DzDof1.6	XM_022916439.1	729	242	24.66	8.41
DzDof2.1	XM_022896728.1	870	289	31.97	6.88
DzDof2.2	XM_022874863.1	957	318	34.01	9.41
DzDof2.4	XM_022888412.1	1029	342	35.97	8.73
DzDof2.5	XM_022903860.1	840	279	30.53	9.27
DzDof3.1	XM_022865170.1	750	249	27.08	8.74
DzDof3.4	XM_022918231.1	714	237	24.97	9.27
DzDof3.5	XM_022889354.1	840	279	30.68	6.70
DzDof3.6	XM_022908803.1	1014	337	36.07	9.08
DzDof3.7	XM_022895569.1	987	328	34.98	8.85
DzDof4.5	XM_022921611.1	948	315	34.48	9.65
DzDof4.6	XM_022877869.1	873	275	30.19	8.46
DzDof5.1	XM_022891906.1	1029	342	35.91	9.45
DzDof5.3	XM_022868588.1	987	328	35.68	9.46
DzDof5.4	XM_022920120.1	945	314	34.26	6.82
DzDof5.6	XM_022881477.1	915	304	33.87	6.25
DzDof5.7	XM_022885261.1	981	326	35.36	9.02

### Phylogenetic analysis of DzDofs

**Figure 2:**
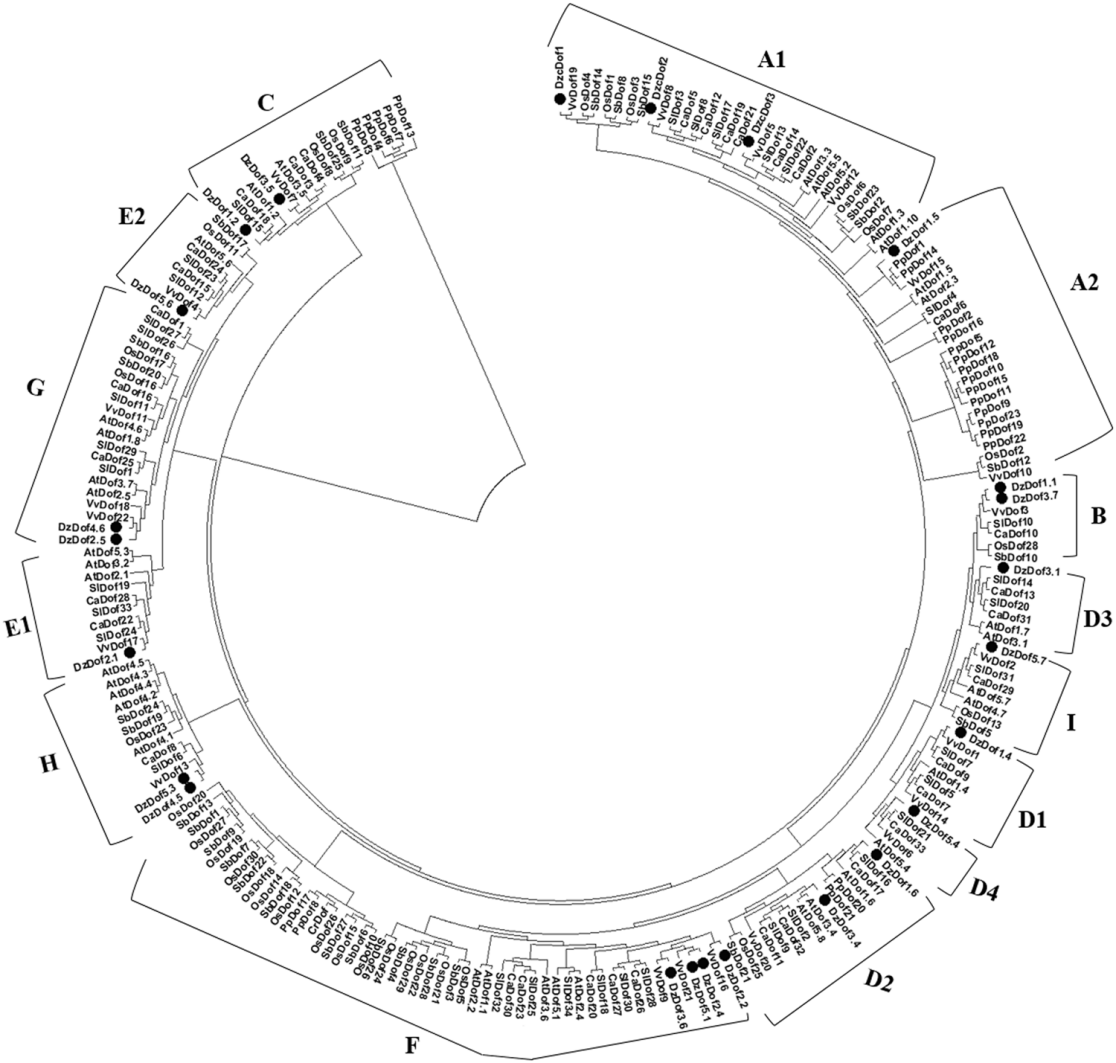
Phylogenetic tree of Dof gene families. Durian DzDofs were aligned with the Dof gene families of eight other representative species (dicots: *Arabidopsis thaliana*, *Solanum lycopersicum*, *Capsicum annuum*, and *Vitis vinifera*; monocots: *Oryza sativa* and *Sorghum bicolor*; non-vascular species: *Chlamydomonas reinhardtii* and *Physcomitrella patens* subsp. *patens*). The full-length protein sequences with 1000 bootstrap replicates were aligned with MEGA X software (with a JTT model and pairwise gap deletion) to construct the phylogenetic tree, using a bootstrap test of phylogeny with the minimum evolution test and default parameters^43^.

To investigate the evolutionary relationships among DzDof TFs and those from eight other representative species (dicots: *Arabidopsis thaliana*, *Solanum lycopersicum*, *Capsicum annuum*, and *Vitis vinifera*; monocots: *Oryza sativa* and *Sorghum bicolor*; non-vascular species: *Chlamydomonas reinhardtii* and *Physcomitrella patens* subsp. *patens*), a neighbour-joining (NJ) phylogenetic tree was constructed by aligning their full-length protein sequences, with 1000 bootstrap replicates. The phylogenetic tree divided the DzDof homologues into nine groups (A to I), which could be further classified into 14 subgroups: A1, A2, B, C, D1, D2, D3, D4, E1, E2, F, G, H, and I (Fig. 2). Group A was the largest clade, containing 56 members, while group B was the smallest clade and harboured only seven members. Interestingly, most of the Dofs from *O*. *sativa* and *S*. *bicolor* were placed in group F, highlighting the fact that these two species belonged to the grass family. Moreover, the only Dof TF from *C*. *reinhardtii* was classified into group F. Many Dofs from *S*. *lycopersicum* and *C*. *annuum* were clustered as pairs, which might point to some levels of domain consensus before the divergence of tomato and pepper. Fourteen pairs of DzDofs with *V*. *vinifera* Dofs (VvDofs) were shown while only one pair of DzDof with *Arabidopsis* Dof (AtDof) was found. All durian cyclic Dofs (DzcDofs) were grouped together in subclade A1.

### Conserved motifs and gene structure analysis

Gene structure analysis revealed no introns in all *DzDofs* (Supplementary Fig. S1). This structural feature (having few (0–2) introns) might be correlated with the important functions of *Dof*, as similar findings were previously reported for *Arabidopsis* and rice Dofs^9^. Moreover, to gain better insights into the conserved motifs of DzDofs, the MEME program was used to analyse putative motifs. As shown in Supplementary Fig. S2, ten distinct motifs were identified. Motif 1 (50 amino acids), known as the Dof domain, was detected at the N-terminus of all DzDofs. All members clustered into one group showed similar motif compositions, which suggested functional similarities among the members of the same group (Supplementary Fig. S3).

### Tissue-specific expression of *DzDofs*

Analysing publicly available transcriptome data from different tissues of the durian cv. Musang King revealed a total of 15 *DzDofs*, including *DzcDof1*, *DzcDof2*, *DzcDof3*, *DzDof1*.*4*, *DzDof1*.*5*, *DzDof2*.*1*, *DzDof2*.*2*, *DzDof2*.*4*, *DzDof2*.*5*, *DzDof3*.*1*, *DzDof4*.*6*, *DzDof5*.*1*, *DzDof5*.*3*, *DzDof5*.*4*, and *DzDof5*.*6*, which were expressed in the fruit pulp at ripe stage (Supplementary Fig. S4). The remaining nine *DzDofs* (*DzDof1*.*1*, *DzDof1*.*2*, *DzDof1*.*6*, *DzDof3*.*4*, *DzDof3*.*5*, *DzDof3*.*6*, *DzDof3*.*7*, *DzDof4*.*5*, and *DzDof5*.*7*) were not expressed in the fruit pulp at ripe stage. However, their expressions could be detected in the other tissues (Supplementary Fig. S4).

### Differential expression patterns of *DzDof*s during post-harvest ripening of the Monthong cultivar

**Figure 3:**
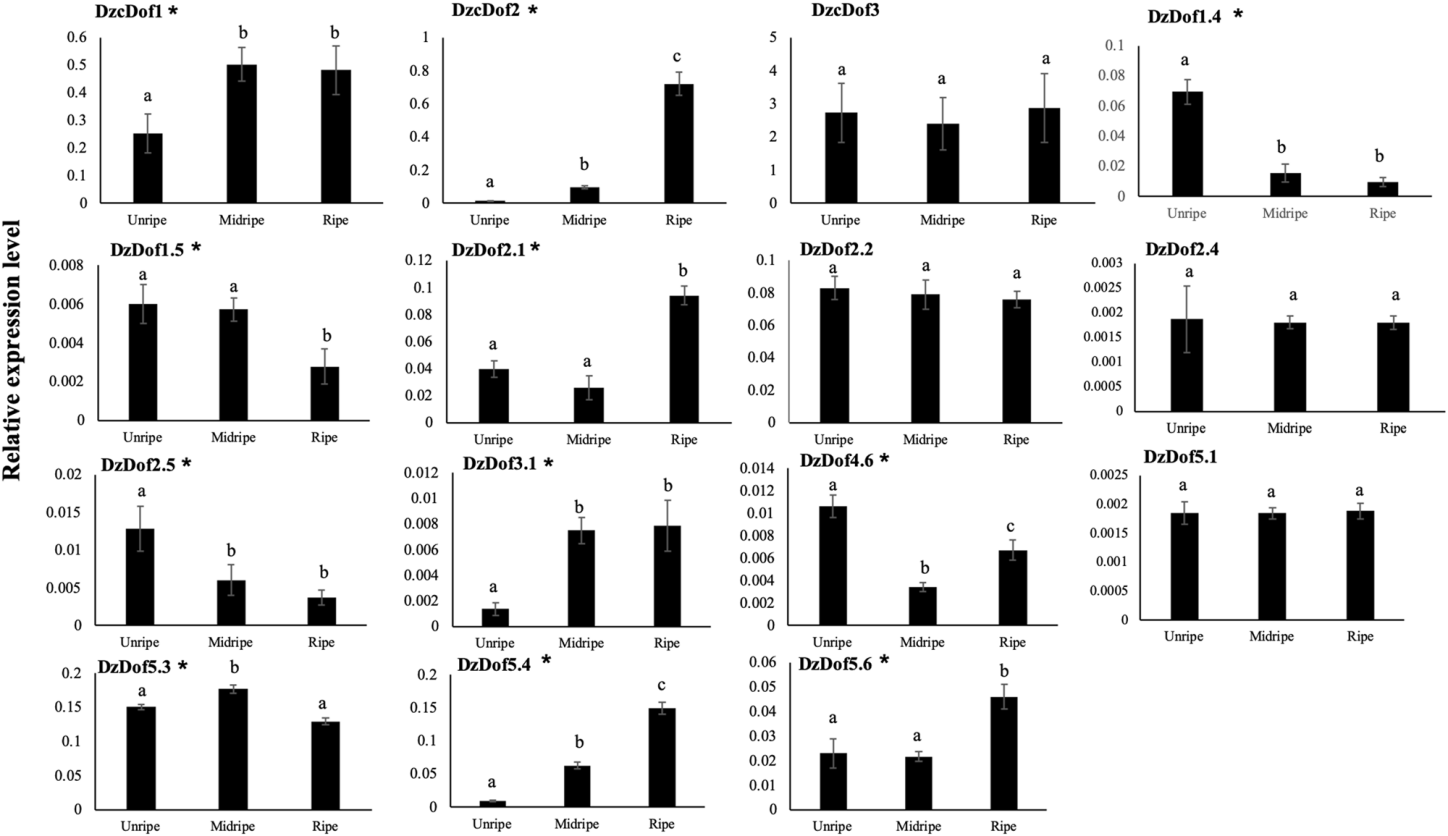
Expression levels of *DzDofs* in durian pulp at three different stages (unripe, midripe, and ripe) during post-harvest ripening in the Monthong cultivar. Gene transcript level was quantified using qRT-PCR and was normalized with respect to the expression level of elongation factor 1 alpha (*EF-1α*), which was used as the reference gene. Error bars represent ± standard deviations (SD). For each *DzDof*, comparisons were made among its expression levels at three different stages. Three independent biological replicates were used. Three technical replicates were also performed for each biological replicate. Bars with different letters above them differed significantly according to Duncan’s multiple range test (*P* < 0.05). Eleven putative ripening-associated *DzDofs* are marked with asterisks (*).

We examined the expression levels of 24 *DzDofs* at three different stages (unripe, midripe, and ripe) during post-harvest ripening. Using qRT-PCR, we observed that 15 of the 24 *DzDofs* were expressed in the fruit pulp, which were: *DzcDof1*, *DzcDof2*, *DzcDof3*, *DzDof1*.*4*, *DzDof1*.*5*, *DzDof2*.*1*, *DzDof2*.*2*, *DzDof2*.*4*, *DzDof2*.*5*, *DzDof3*.*1*, *DzDof4*.*6*, *DzDof5*.*1*, *DzDof5*.*3*, *DzDof5*.*4*, and *DzDof5*.*6* (Fig. 3). This finding was similar to the fruit pulp-expressed *DzDofs* in Musang King cultivar. Notably, these 15 fruit pulp-expressed *DzDofs* showed different expression patterns during post-harvest ripening of durian fruit. Out of the 15 fruit pulp-expressed *DzDofs*, the expression patterns of 11 varied over the course of post-harvest ripening. Six *DzDofs*, including *DzcDof1*, *DzcDof2*, *DzDof2*.*1*, *DzDof3*.*1*, *DzDof5*.*4*, and *DzDof5*.*6*, were expressed at increasingly high levels as fruit ripening progressed in the Monthong cultivar. Moreover, four *DzDofs*, including *DzDof1*.*4*, *DzDof1*.*5*, *DzDof2*.5, and *DzDof4*.*6*, were expressed at decreasing levels over the course of fruit ripening. In addition, *DzDof5*.*3* was expressed at significantly higher levels at the midripe compared to the unripe stage. However, its expression level declined from the midripe to the ripe stage. Taken together, these 11 *DzDofs* showed ripening-associated expression patterns, and their expression levels were further examined under ethylene treatment. In contrast, the expression of *DzcDof3*, *DzDof2*.*2*, *DzDof2*.*4*, and *DzDof5*.*1* did not vary significantly during post-harvest ripening, and thus these were not considered to be ripening-associated TFs.

### Validation of ripening-associated *DzDofs* under three ripening treatments of the Monthong cultivar

**Figure 4:**
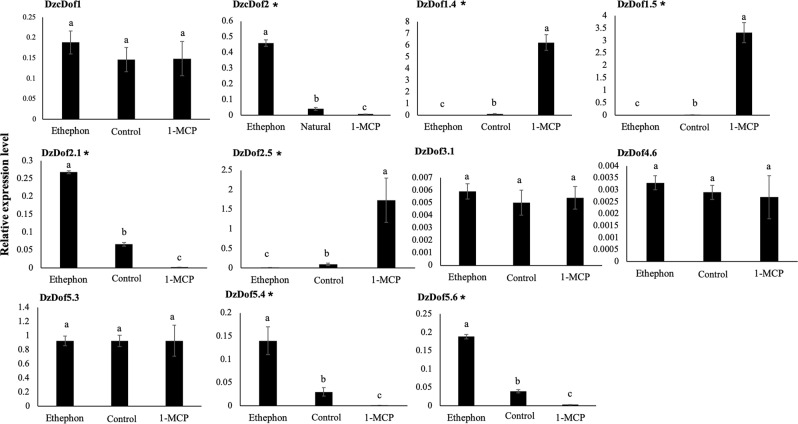
Expression levels of 11 putative ripening-associated *DzDofs* under three different ripening treatments: natural (control), ethylene-induced, and 1-MCP-delayed ripening. Gene transcript level was quantified using qRT-PCR. Elongation factor 1 alpha (*EF-1α*) was used as the reference gene to normalize the expression levels of each other gene. Error bars indicate ± standard deviations (SD). For each *DzDof*, comparisons were made of its expression levels among three different ripening treatments. Three independent biological replicates were used. Three technical replicates were also performed for each biological replicate. Bars with different letters above them were significantly different according to Duncan’s multiple range test (*P* < 0.05). The *DzDofs* marked with asterisks (*) indicate the seven confirmed ripening-associated *DzDofs*.

Ethylene plays a major role in climacteric fruit ripening. To further confirm the expression levels of the putative ripening-associated *DzDofs* under ethylene treatment, their transcript levels were measured under three different ripening conditions: natural, ethephon-induced, and 1-methylcyclopropene (1-MCP)-delayed ripening. As shown in Fig. 4, the expression levels of *DzcDof2*, *DzDof2*.*1*, *DzDof5*.*4*, and *DzDof5*.*6* were significantly enhanced under ethylene treatment compared to those during natural ripening (control). However, under 1-MCP treatment, their transcript levels were dramatically decreased compared to those during natural ripening. Moreover, expression levels of *DzDof1*.*4*, *DzDof1*.*5*, and *DzDof2*.*5* were dramatically suppressed under ethylene treatment, whereas 1-MCP treatment significantly enhanced their expression levels relative to those during natural ripening. These results provide convincing evidence regarding the ripening-associated roles of these seven *DzDofs* during fruit ripening. However, the expression of *DzcDof1*, *DzDof3*.*1*, *DzDof4*.*6*, and *DzDof5*.*3* did not vary significantly under different ripening treatments, and therefore these were not considered to be ripening-associated TFs.

### Differential expression levels of fruit pulp-expressed *DzDofs* during post-harvest ripening of the Chanee cultivar

To examine the expression levels of *DzDofs* in another cultivar with different ripening behaviour and sensory characteristics, their expression levels were also measured during post-harvest ripening in the Chanee cultivar. The expression levels of *DzDofs* in Chanee followed a similar pattern to those in Monthong (Supplementary Fig. S5). *DzcDof2*, *DzDof2*.*1*, *DzDof5*.*4*, and *DzDof5*.*6* were expressed at increasingly high levels during fruit ripening in the Chanee cultivar. Moreover, *DzDof1*.*4*, *DzDof1*.*5*, and *DzDof2*.*5* were expressed at decreasing levels during ripening in this cultivar.

To further explore any potential association between the expression profiles of *DzDofs* and the different ripening characteristics of the Monthong and Chanee cultivars, the expression levels of Monthong *DzDofs* were compared to those in the Chanee cultivar. Among the 15 fruit pulp-expressed *DzDofs*, the expression profiles of 10 *Dofs*, including *DzcDof2*, *DzDof1*.*4*, *DzDof2*.*1*, *DzDof2*.*2*, *DzDof2*.*5*, *DzDof3*.*1*, *DzDof4*.*6*, *DzDof5*.*3*, *DzDof5*.*4*, and *DzDof5*.*6*, showed significant differences between the two cultivars in at least one stage of ripening (Supplementary Fig. S5). These ten *DzDofs* were thus considered potential cultivar-dependent TFs. However, the expression levels of the remaining *DzDofs* did not show any significant differences between the two cultivars during ripening. Among the cultivar-dependent *DzDofs*, *DzDof5*.*4* had the greatest fold increase at the ripe stage in Chanee compared with that in Monthong. Moreover, *DzDof2*.*2* was the only Dof whose expression level was higher in Chanee, and it showed a significant fold difference between the two cultivars at each ripening stage. This *DzDof* might be responsible for the different ripening characteristic of Chanee and Monthong, since its expression levels were significantly higher at each ripening stage in the Chanee cultivar than those in the Monthong cultivar. Hence, *DzDof2*.*2* was further selected for functional prediction analysis via orthologue identification.

### Orthologue identification and gene function prediction of the candidate cultivar-dependent DzDof2.2

Characterized orthologue identification is widely used as a powerful tool for functional prediction in genes^17–19^. It has been shown that orthologous proteins have similar biological functions in different species^17^. The protein sequence of DzDof2.2 was used as a query to find orthologous proteins in *Arabidopsis*. As a result, AtDof2.4, AtDof5.1, AtDof3.6 and AtDof1.1 (OBP2) were found to be the orthologues of DzDof2.2. This was consistent with the phylogenetic tree (Fig. 2), which clustered DzDof2.2 with the mentioned AtDofs together in the clade F. According to the functional characterization of AtDof1.1^13^, significantly higher auxin levels were measured in the leaves and seedlings of AtDof1.1 over-expressing lines compared to those in wild-type plants. Accordingly, it might be possible that DzDof2.2 could also regulate auxin biosynthesis during ripening in durian. This finding prompted us to further measure endogenous auxin levels in the fruit pulps of both cultivars at different stages of ripening.

### Higher indole-3-acetic acid (IAA) and auxin biosynthetic gene expression levels during the ripening of Chanee compared to Monthong

**Figure 5:**
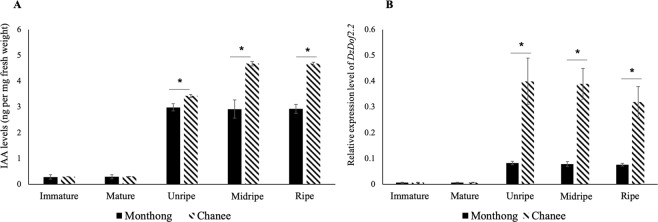
Indole-3-acetic acid (IAA) and *DzDof2*.*2* expression levels. (**A**) IAA levels were measured at five different stages (immature, mature, unripe, midripe, and ripe) in both the Monthong and Chanee cultivars. Comparisons were made between the two cultivars at each stage. An asterisk (∗) above the bars indicates a significant difference between cultivars (Student’s *t*-test, *P* < 0.05). (**B**) Expression levels of *DzDof2*.*2* were measured at five different stages (immature, mature, unripe, midripe, and ripe) in both the Monthong and Chanee cultivars. Comparisons were made between the two cultivars at each stage. Three independent biological replicates were used. Three technical replicates were also performed for each biological replicate. An asterisk (∗) above the bars indicates a significant difference between cultivars (Student’s *t*-test, *P* < 0.05).

Since the cultivar-dependent DzDof2.2 could play a role in ripening by regulating auxin biosynthesis, endogenous levels of IAA were measured in the pulps of durian fruits at five different stages before (immature and mature) and during post-harvest ripening (unripe, midripe, and ripe) of both the Monthong and Chanee cultivars. As shown in Fig. 5A, during the immature and mature stages, IAA was detected at low levels, which were not significantly different between the two cultivars. However, after being harvested (from the mature to unripe stages), IAA levels significantly increased in both cultivars, with there being a significantly greater increase in Chanee than in Monthong. Moreover, IAA levels were significantly higher during the post-harvest ripening of Chanee compared to those in Monthong. The higher IAA levels in Chanee could contribute to its faster post-harvest ripening compared to that of Monthong through enhancing climacteric ethylene biosynthesis (auxin-ethylene crosstalk). Assessing transcript levels by qRT-PCR confirmed the higher expression levels of both ethylene biosynthetic genes, e.g. *ACC synthase* (*ACS*) and *ACC oxidase* (*ACO*), during ripening of Chanee compared to Monthong (Supplementary Fig. S6), suggesting a higher endogenous ethylene content in Chanee. Moreover, the expression levels of both genes peaked at the ripe stage, indicating the highest levels of climacteric ethylene biosynthesis at this stage during post-harvest ripening of durian fruit (Supplementary Fig. S6).

To assess the correlation between *DzDof2*.*2* expression levels and IAA content, we analysed the expression levels of *DzDof2*.*2* before ripening at the immature and mature stages, in addition to the previous results assessed during post-harvest ripening (Supplementary Fig. S5), in both cultivars. At these two stages, it was found that *DzDof2*.*2* was expressed at low levels in both the Monthong and Chanee cultivars, without any significant difference between the two cultivars (Fig. 5B). However, there was a significant increase in the expression levels of *DzDof2*.*2* from the mature to the unripe stage in both cultivars, with a significantly greater fold change in Chanee compared to that in Monthong (Fig. 5B). The consistency of the expression levels of *DzDof2*.*2* with auxin levels before and during ripening could support the possible role of *DzDof2*.*2* in controlling auxin biosynthesis.

**Figure 6:**
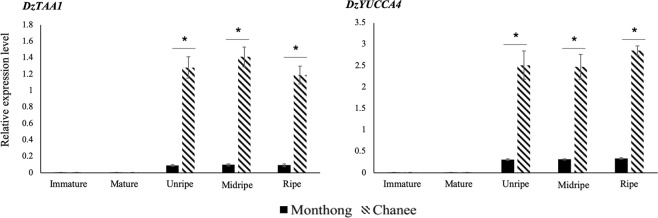
Expression levels of auxin biosynthetic genes. Expression levels of two candidate auxin biosynthetic genes, L-tryptophan aminotransferase 1 (*TAA1*) (left panel) and the indole-3-pyruvate monooxygenase (*YUCCA4*) (right panel) of the indole-3-pyruvic acid (IPyA) pathway, were measured at five different stages (immature, mature, unripe, midripe, and ripe) in both the Monthong and Chanee cultivars. Comparisons were made between the two cultivars at each stage. Three independent biological replicates were used. An asterisk (∗) above the bars indicates a significant difference between cultivars (Student’s *t*-test, *P* < 0.05).

From the analysis of our in-house transcriptome data of Chanee fruit pulp, we identified two candidate auxin biosynthetic genes, including L-tryptophan aminotransferase 1 (*TAA1*) and the indole-3-pyruvate monooxygenase (*YUCCA4*) of the indole-3-pyruvic acid (IPyA) pathway. Interestingly, *TAA1* and *YUCCA4* had low expression levels during the immature and mature stages (before post-harvest ripening), with no significant differences between the two cultivars (Fig. 6). However, there was a dramatic increase in the expression levels of both genes after harvesting (from the mature to the unripe stage) in both the Monthong and Chanee cultivars. This phenomenon was consistent with the increased auxin levels observed after harvesting, which could occur as a stress response to mechanical injury during fruit harvesting. Notably, *TAA1* and *YUCCA4* were expressed at significantly higher levels during the post-harvest ripening of Chanee compared to that of the Monthong cultivar (Fig. 6). This observation was consistent with the higher IAA levels observed during ripening in the Chanee than in the Monthong cultivar. Interestingly, we scanned the promoter regions of *TAA1* and *YUCCA4* and identified 40 and 34 cis-regulatory motifs (AAAG or its reverse-oriented sequence CTTT, known as the binding site for Dof TFs)^1,2^, respectively (Supplementary Fig. S7). This observation could pinpoint the possible role of Dof TFs in the transcriptional regulation of auxin biosynthetic genes.

#### Functional characterization of *DzDof2*.*2* through transient expression in *Nicotiana benthamiana*

**Figure 7:**
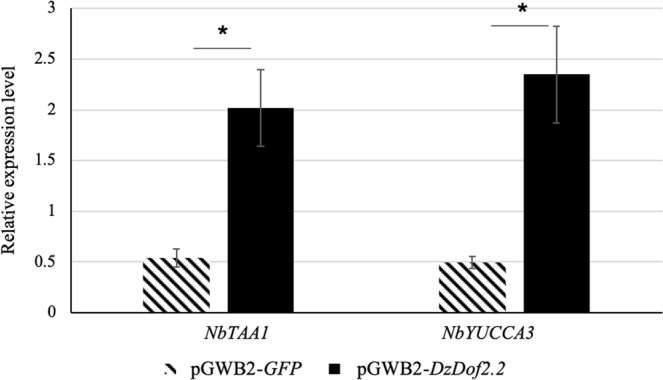
Expression levels of auxin biosynthetic genes in *Nicotiana benthamiana* transiently expressing *DzDof2*.*2*. Expression levels of two candidate auxin biosynthetic genes from *N*. *benthamiana*, L-tryptophan aminotransferase 1 (*NbTAA1*) and the indole-3-pyruvate monooxygenase (*NbYUCCA3*) of the indole-3-pyruvic acid (IPyA) pathway, were measured in *N*. *benthamiana* leaves infiltrated with pGWB2-*DzDof2*.*2* (treatment) and pGWB2-*GFP* (control). Comparisons were made between the control and treatment. Three independent biological replicates were used. An asterisk (∗) above the bars indicates a significant difference (Student’s *t*-test, *P* < 0.05).

To assess the function of *DzDof2*.*2 in planta*, it was transiently expressed in the leaves of *N*. *benthamiana* and the expression levels of major auxin biosynthetic genes from *N*. *benthamiana*, *NbTAA1* and *NbYUCCA3*, were analysed. We observed a significantly higher expression levels of both *NbTAA1* and *NbYUCCA3* in the leaves transiently expressing *DzDof2*.*2* when compared to the control (Fig. 7). This observation confirmed the transcriptional regulation of auxin biosynthetic genes by DzDof2.2 TF. Consistently, our *in silico* analysis identified cis-regulatory binding sites (AAAG/CTTT) for Dof TFs in the promoter regions of both *NbTAA1* and *NbYUCCA3* (Supplementary Fig. S8).

## Discussion

TFs play significant roles in regulating gene expression networks. A large body of evidence suggests that the identification and functional characterization of TFs should provide a better understanding of how plants develop, grow, and respond to environmental stimuli. Herein, we conducted a genome-wide analysis and identified 24 putative *DzDofs* in the durian genome (Musang King cv.). The number of identified *DzDofs* was lower than that found in banana (74 MaDof)^11^, rice (30 OsDof)^20^, *Arabidopsis* (36 AtDof)^2^, tomato (34 SiDof)^5^, Chinese cabbage (76 BraDof)^21^, cucumber (36 CsDof)^9^, and potato (35 StDof)^4^. Consistent with previous studies, the alignment of DzDof protein sequences revealed a highly conserved Dof domain, which was observed at the N-terminal regions of all 24 DzDofs (Fig. 1), suggesting that Dof TFs have been evolutionarily conserved among plants. Apart from the Dof domain, nine other distinct motifs were identified that were differentially distributed among DzDofs. In addition, as predicted by *in silico* analyses, all of the 24 deduced DzDofs harboured NLSs to help localize them to the nucleus. Digital expression profiles of *DzDofs* from Musang King cultivar revealed a total of 15 ripe fruit pulp-expressed *Dofs* (Supplementary Fig. S4). These 15 *DzDofs* were identical to those expressed during post-harvest ripening of both Monthong and Chanee cultivars, suggesting their functions in the pulp tissue. Dof TFs have been shown to play crucial roles in various physiological phenomena, such as the regulatory networks of plant responses to diverse biotic and abiotic stressors, seed germination, morphology, light responses, and fruit ripening^3,11,22^.

In our study, among the 15 fruit pulp-expressed *Dofs* in Monthong cultivar, seven ripening-associated *DzDofs*, including *DzcDof2*, *DzDof1*.*4*, *DzDof1*.*5*, *DzDof2*.*1*, *DzDof2*.*5*, *DzDof5*.*4*, and *DzDof5*.*6*, were identified based on their differential expression during post-harvest ripening in the Monthong cultivar (Fig. 3). The expression levels of *DzcDof2*, *DzDof2*.*1*, *DzDof5*.*4*, and *DzDof5*.*6* were confirmed to be induced under ethephon and suppressed under 1-MCP treatment (Fig. 4). These ethylene-inducible *DzDofs* were expressed at increasing levels during ripening, and thus could act as either putative transcriptional activators or repressors of ripening. Zhang *et al*.^12^ identified an ethylene-induced Dof TF in kiwifruit (AdDof3) that acted as a ripening-activator TF and enhanced starch degradation during fruit ripening. On the other hand, an ethylene-induced banana Dof (MaDof23), which was expressed at increasing levels during ripening, was a transcriptional repressor. This Dof acted antagonistically to a ripening-activator ethylene response factor (MaERF9) to regulate banana fruit ripening^11^. Notably, the expression levels of the remaining ripening-associated *DzDofs* (*DzDof1*.*4*, *DzDof1*.*5*, and *DzDof2*.*5*) were dramatically suppressed under ethephon and induced under 1-MCP treatment. It is interesting that these ripening-associated *DzDofs* were expressed at decreasing levels during ripening. To the best of our knowledge, this is the first report describing this particular effect of 1-MCP treatment on Dof TFs. The 1-MCP treatment delays climacteric ripening by inhibiting ethylene-dependent signalling by competitively binding to ethylene receptors^23,24^. Besides this inhibitory effect, 1-MCP also downregulates the expression of ethylene biosynthetic genes^25–27^ (e.g., 1-aminocyclopropane-carboxylase (ACC) synthase (*ACS*) and ACC oxidase (*ACO*)), ethylene perception genes^27–30^ (e.g., ethylene receptors *ETR* and *ERS*), and ethylene signalling genes^31,32^ (e.g., ethylene response factor (*ERF*)). It is possible that 1-MCP upregulates the expression of these three *DzDofs* by inhibiting ethylene biosynthesis and signalling. This observation suggests that these *DzDofs* could be negatively regulated by ethylene, and act as putative transcriptional repressors of ripening. Consistent with our observations, the expression of a kiwifruit Dof (AdDof4) was suppressed by ethylene treatment and was thus considered to be a ripening-repressor TF^12^.

In addition, we compared the expression patterns of *DzDofs* between the two cultivars and found that *DzDofs* in the Chanee cultivar showed similar expression patterns, including identical ripening-associated *DzDofs*, to those in the Monthong cultivar. The similar expression patterns of *DzDofs* between Monthong and Chanee cultivars suggest that these *DzDofs* might have similar regulatory roles during fruit ripening in these two cultivars. However, although the expression patterns were similar between the two cultivars, comparisons of the expression levels of fruit pulp-expressed *DzDofs* between cultivars revealed a total of 10 potential cultivar-dependent Dofs. Among these, the expression levels of *DzDof2*.*2* underwent a significantly greater fold change at each ripening stage in the Chanee than in the Monthong cultivar. Although the expression level of *DzDof2*.*2* did not vary significantly during the unripe, midripe, and ripe stages, its expression level harboured a significant increase from mature (before ripening) to unripe stage (post-harvest ripening) in both cultivars with a greater fold increase in Chanee than in Monthong (Fig. 5B). Therefore, we hypothesized that *DzDof2*.*2* would play a role during ripening of durian fruit and the difference in its expression levels between the two cultivars might contribute to the faster ripening and different sensory characteristics of the Chanee cultivar from those of the Monthong one.

To examine the possible function of DzDof2.2, we found its orthologues in *Arabidopsis*. Among the four identified orthologues, *AtDof1*.*1* (OBP2) is involved in regulating glucosinolate biosynthesis. In addition, transgenic *Arabidopsis* lines over-expressing OBP2 had higher auxin concentrations in their leaves and seedlings compared to those in the wild type^13^. This phenomenon could suggest a role of *DzDof2*.*2* in regulating auxin biosynthesis. Higher expression levels of *DzDof2*.*2* could enhance auxin biosynthesis, leading to the higher auxin levels during ripening in the Chanee than in the Monthong cultivar (Fig. 5A). Notably, the increased expression levels of *DzDof2*.*2* were consistent with the increased auxin levels from the mature to the unripe stage (Fig. 5). We further confirmed that the higher level of auxin in Chanee co-occurred with the increased expression of auxin biosynthetic genes. Four tryptophan-dependent auxin biosynthetic pathways have been well-characterized in *Arabidopsis*, including the indole-3-pyruvic acid (IPyA), tryptamine (TAM), indole-3-acetaldoxime (IAOx), and indole-3-acetamide (IAM) pathways^33^. Among these pathways, IPyA is considered the major auxin biosynthetic pathway in many plant species^34^. The main biosynthetic enzymes of the IPyA pathway in durian, *DzTAA1* and *DzYUCCA4*, were expressed at significantly higher levels during post-harvest ripening of the Chanee cultivar compared to those in the Monthong cultivar. Moreover, there was a significant increase in the expression levels of both genes from the mature to the unripe stage, with a higher fold increase in Chanee compared to that in Monthong (Fig. 6). This expression profile was consistent with the changes in expression levels of *DzDof2*.*2* and IAA levels. It has been well-documented that auxin plays a major role during the ripening of climacteric fruits, such as peach, tomato, and papaya^35,36^. Concomitant with the increase in climacteric ethylene biosynthesis during ripening of tomato and peach, elevated levels of auxin have also been measured^35^. Auxin can stimulate climacteric ethylene biosynthesis through upregulating the ACC synthase gene family (auxin-ethylene crosstalk)^37–39^. Consistently, we observed significantly higher expression levels of both ethylene biosynthetic genes (*ACS* and *ACO*) during post-harvest ripening of Chanee compared to Monthong, suggesting higher ethylene content in Chanee than in Monthong. It has been well documented that the expression levels of ethylene biosynthetic genes are positively correlated with the ethylene content during climacteric ripening^40,41^. Besides this well-known role of auxin in upregulating ethylene biosynthesis, auxin can also play a role of its own during climacteric ripening by transcriptionally modulating other ripening-related auxin-responsive genes^35^. Taken together, our results demonstrate that auxin is actively involved in durian fruit ripening.

Interestingly, the promoter regions of *DzTAA1* and *DzYUCCA4* were enriched with cis-regulatory binding sites (AAAG/CTTT) for Dof TFs. This observation could suggest the possible transcriptional regulation of these two genes by Dof TFs. Our results obtained from transient expression of *DzDof2*.*2* in *N*. *benthamiana* leaves provided convincing evidence of the possible role of *DzDof2*.*2* in transcriptional regulation of auxin biosynthetic genes during ripening in durian (Fig. 7). Consistent with the promoters of auxin biosynthetic genes from durian, we identified the occurrences of Dof TF binding sites in the promoter regions of *NbTAA1* and *NbYUCCA3* (Supplementary Fig. S8).

It is notable that, among the 10 identified cultivar-dependent *DzDofs*, *DzDof5*.*4* had the greatest fold increase at the ripe stage in Chanee relative to that in Monthong (Supplementary Fig. S5). OBP4 (AtDof5.4) was the closest orthologue of DzDof5.4. Interestingly, exogenous IAA induced the expression of *AtDof5*.*4*. This might explain why the expression levels of *DzDof5*.*4* peaked at the ripe stage in both cultivars, with a dramatically greater fold increase in Chanee compared to that in Monthong. The higher IAA levels during ripening, the greater expression levels of *DzDof5*.*4* were thus observed in our study.

To summarize, here, we reported a comprehensive analysis of durian Dof TFs. Out of 24 identified *DzDofs*, 15 were fruit pulp-expressed, among which seven *DzDofs* were identified as being ripening-associated for both the Chanee and Monthong cultivars. Notably, comparing the expression patterns of fruit pulp-expressed *DzDofs* between the Monthong and Chanee cultivars revealed 10 potential cultivar-dependent TFs, among which *DzDof2*.*2* showed a significantly greater fold increase at every ripening stage in Chanee than in Monthong. We suggest that DzDof2.2 could transcriptionally control the expression of auxin biosynthetic genes. Higher expression levels of DzDof2.2 in Chanee could enhance its auxin levels during ripening through transcriptional regulation of auxin biosynthetic genes. Higher auxin levels in Chanee would upregulate ethylene biosynthesis by transcriptionally activating the ACC synthase gene family, and lead to an earlier ethylene response (auxin-ethylene crosstalk), and thus faster post-harvest ripening compared to that in the Monthong cultivar. Our results provide compelling evidence of the role of Dof TFs during the post-harvest ripening of durian. The results obtained herein should provide an important foundation for future studies involving gene cloning and the functional characterization of ripening-associated and cultivar-dependent DzDofs.

## Materials and Methods

### Plant materials and treatments

Durian (*Durio zibethinus* Murr.) cv. Monthong and Chanee fruits were collected from a commercial orchard located in Trat province in the eastern part of Thailand in early April 2017. Fruit samples of similar size and weight (~3–4 kg each) were harvested at two different stages: immature (at 70 days (for Chanee) and 85 days (for Monthong) after anthesis) and mature (at 90 days (for Chanee) and 105 days (for Monthong) after anthesis). Immature samples were peeled immediately after harvesting, whereas some mature samples were peeled immediately, and some were kept at room temperature (30 °C) until peeling. Five types of samples were used in this study: immature, mature, unripe (harvested at the mature stage and kept at room temperature for one day (both cultivars) and then peeled), midripe (harvested at the mature stage and kept at room temperature for two days (Chanee) or three days (Monthong) and then peeled), and ripe (harvested at the mature stage and kept at room temperature for four days (Chanee) or five days (Monthong) and then peeled). After peeling, two of the central pulps were collected from each fruit sample, following the method described by Pinsorn *et al*.^15^. Briefly, the first pulp was collected along with a seed, and was used to measure fruit firmness with a texture analyser (TA-XT2i; Stable Micro Systems, Godalming, UK) to assure that samples of the two cultivars were compared at the same ripening stages. A puncture test with a 6-mm probe, at a test speed of 2 mm/s and testing distance of 5 mm, was carried out on five random points in each pulp. Midripe and ripe pulps had a mean ± SD firmness of 3.4 ± 0.81 and 1.55 ± 0.45 N, respectively, in both cultivars. After this test, the second pulp was collected without a seed, immediately frozen in liquid nitrogen, and stored at −80 °C for further use.

To further investigate the role of ethylene during post-harvest ripening, three different ripening treatments were applied as follows: natural, ethephon-induced, and 1-methylcyclopropene (1-MCP)-delayed ripening. Mature durian samples were collected and treated with either ethephon (48% 2-chloroethylphosphonic acid; Alpha Agro Tech Co., Ltd., Thailand) or 1-MCP (0.19% 1-MCP tablet; BioLene Co., Ltd., China) for ethephon-induced and 1-MCP-delayed ripening, respectively. For ethephon treatment, the ethephon solution was applied to the upper area of each fruit stalk. For 1-MCP treatment, each fruit was placed inside a closed 20-L chamber. Then, one tablet of 1-MCP was placed into a beaker inside the chamber. Water (5 mL) was added to the beaker to generate gaseous 1-MCP and the chamber was immediately closed for 12 h at room temperature (30 °C) while the control samples were kept under similar conditions without 1-MCP. After treatment, control and treated samples were kept at room temperature (30 °C) until the ethephon-treated samples ripened. All samples from the three ripening conditions were then peeled. After that, two central pulps were collected from each sample and processed as mentioned previously. In this study, for each type of sample (immature, mature, unripe, midripe, and ripe, as well as natural ripening, ethephon-induced ripening, and 1-MCP-delayed ripening), three biological replicates were used. Each biological replicate was defined as one durian fruit harvested from a separate tree.

For the agroinfiltration experiment, *N*. *benthamiana* seeds were sown in pots containing peat moss and were grown under controlled conditions (temperature 25 °C and 16/8 h light/dark photoperiod; artificial light of 4,500 Lux). Two-week-old plants were transplanted individually into pots and were grown under similar conditions.

### *In silico* identification and analysis of durian Dofs

The whole-genome sequence of durian cv. ‘Musang King’ was used to identify putative durian Dofs. First, the conserved Dof domain (PF02701) determined based on a Hidden Markov Model (HMM) was obtained from the Pfam protein database (http://pfam.xfam.org). The HMM profile of the Dof domain was then used as a query to search against in the Musang King genome database^16^. The amino acid sequences of DzDofs were further obtained and checked for the presence of the highly conserved motif (Dof domain) at the N-terminus. The isoelectric point (*p*I) and molecular weight of each of the identified DzDof proteins were then calculated using the web-based tool ExPASy (http://web.expasy.org/compute_pi/)^42^. Nuclear localization signal (NLS) analysis was also done using a web-based tool, found at: http://cello.life.nctu.edu.tw ^9^.

### Phylogenetic analysis of DzDofs

To investigate phylogenetic relationships among DzDofs and Dofs from eight other representative species (dicots: *Arabidopsis thaliana*, *Solanum lycopersicum*, *Capsicum annuum*, and *Vitis vinifera*; monocots: *Oryza sativa* and *Sorghum bicolor*; non-vascular species: *Chlamydomonas reinhardtii* and *Physcomitrella patens* subsp. *patens*), a neighbour-joining (NJ) phylogenetic tree was constructed by aligning their full-length protein sequences with 1000 bootstrap replicates using MEGA X software (with a JTT model and pairwise gap deletion)^43^.

### Gene structure analysis and identification of conserved motifs

To obtain a deeper understanding of *DzDof* structures, Gene Structure Display Server 2.0 (with default parameters) (http://gsds.cbi.pku.edu.cn) was used to characterize the exon-intron structures of *DzDof-*family genes. Motifs were identified using the MEME program (http://meme-suite.org)^44^, with the following parameter settings: motif length = 6–100; motif sites = 2–120; maximum number of motifs = 10; and the distribution of one single motif was “any number of repetitions”.

### Tissue-specific expression analysis

In order to analyse the expression levels of *DzDofs* in different tissues, Illumina reads were obtained from a public repository database (SRA, Sequence Read Archive) as follows: SRX3188225 (root tissue), SRX3188222 (stem tissue), SRX3188226 (leaf tissue), and SRX3188223 (aril/pulp tissue). FASTQC software was used to analyse the total reads quality parameters (https://www.bioinformatics.babraham.ac.uk/projects/fastqc/). To filter low quality reads, the trimming process was performed using Trimmomatic V0.32 (http://www.usadellab.org/cms/?page=trimmomatic) (https://github.com/seqan/flexbar). Thereafter, transcriptome assembly was performed using Trinityrnaseq-Trinity-v2.4.0 (https://github.com/trinityrnaseq/trinityrnaseq/wiki/Running-Trinity)^45^. For expression profiling analysis, abundance estimation tool in Trinity package was used to align the input reads to the de novo assembled transcriptome to obtain raw counts of each contig. The raw read counts were then merged in a single read count matrix. This matrix was used as input for normalization to generate a Trimmed Mean of M-values (TMM) normalized matrix. Thereafter, the normalized total read counts (RC) were used to generate heatmap using MetaboAnalyst 4.0, an open source R-based program^46^. For the heatmap construction, the values were sum normalized, log2 transformed and autoscaled.

### RNA isolation and quantitative real time polymerase chain reaction (qRT-PCR)

Total RNA was isolated from durian fruit pulp and purified using PureLink Plant RNA Reagent (Thermo Fisher Scientific™) according to the manufacturer’s instructions. Samples were treated with DNase I (Thermo Fisher Scientific™) to remove genomic DNA. The quality and quantity of RNA samples were assessed using agarose gel electrophoresis and an Eppendorf BioPhotometer D30 with A260/280 and A260/230 ratios between 1.8 to 2.0 and 2.0 to 2.2, respectively. One microgram of total RNA was reverse-transcribed to cDNA using a RevertAid First Strand cDNA Synthesis Kit (Thermo Fisher Scientific™), in accordance with the manufacturer’s recommended protocol. All primers used in this study were designed using Primer 3 online (http://primer3.ut.ee/). The primers used are presented in Supplementary Table S2. qRT-PCR was performed to investigate patterns in the expression of *DzDofs* at the immature and mature stages (before ripening) and unripe, midripe and ripe stages (post-harvest ripening) of fruits of both the Monthong and Chanee cultivars. The PCR reaction was performed in a total volume of 20 μL containing 1 μL of diluted cDNA (1 ng of cDNA), 10 μL of 2x QPCR Green Master Mix LRox (biotechrabbit, Berlin, Germany), and 200 nM of each gene-specific primer. Amplification was carried out with a CFX95 Real-time System (Bio-Rad Laboratories Inc., California, USA) under the following conditions: initial activation at 95 °C for 3 min, followed by 40 cycles of denaturation at 95 °C for 15 s, then annealing at 56–60 °C for 30 s, and finally extension at 72 °C for 20 s. A melting curve analysis for testing specific products was carried out by heating the products from 55 °C to 95 °C at increments of 0.5 °C. A single product was obtained for each *DzDof*. All qRT-PCR experiments were performed with three independent biological replicates. The elongation factor 1 alpha (*EF-1α*) gene was used as a reference gene (See Supplementary Table S3 for primers). The relative expression level of each gene compared with that of the reference gene was calculated using the 2^-ΔCt^ method^47^.

### Identification of orthologues

Orthologues of candidate DzDofs in *Arabidopsis* were identified using the web-based tool EnsemblPlants, release 40 (https://plants.ensembl.org/index.html), using the default parameters^48^.

### Indole-3-acetic acid (IAA) measurement

Endogenous IAA levels in fruit pulps from both cultivars were measured. IAA was extracted from fruit pulp samples following the method described by Sinha and Basu^49^. Briefly, each pulp sample was freeze-ground into a fine powder. After this, 500 mg of the powder was mixed with 10 mL of chilled 80% ethanol, vortexed vigorously for 3 min, and kept at 4 °C for 2 h with occasional vortexing (at least five times). Afterwards, the mixture was centrifuged at 10,000 rpm (4 °C) for 15 min, and the supernatant was then used for IAA measurements. The IAA concentration (A_535_) was quantitatively measured via the colorimetric assay of Gordon and Weber^50^ using a standard curve prepared from serial dilutions of commercial IAA stock solutions (Duchefa Biochemie, The Netherlands), ranging from 7.5 to 500 μM and with an *R*^2^ of 0.99.

### *In silico* identification and promoter analysis of auxin biosynthetic genes

Based on the auxin biosynthetic pathways identified in *Arabidopsis*, the indole-3-pyruvic pathway was selected as the candidate pathway for auxin biosynthesis^34^ in durian. The conserved domain of each biosynthetic enzyme (based on a HMM) was obtained from the Pfam protein database (http://pfam.xfam.org/) as follows: using the PF00155 for aminotransferase (TAA1) and the PF00743 for flavin-containing monooxygenases of the YUCCA family (YUCCA4). The HMM profile of each enzyme was then used as a query to search against our in-house transcriptome database and the Musang King genome (i.e., TAA1 (XM_022878297.1) and YUCCA4 (XM_022900772.1)). For qRT-PCR, primers were designed as mentioned previously (Supplementary Table S3). In order to analyse cis-regulatory elements in the promoter regions of auxin biosynthetic genes, the 2000-bp upstream promoter regions of these genes were scanned for the Dof domain (AAAG/CTTT) using the online tool PLACE (http://www.dna.affrc.go.jp/)^36^.

### Expression analysis of *ACC synthase* (*ACS*) and *ACC oxidase* (*ACO*) in Monthong and Chanee cultivars

In order to compare the expression levels of ethylene biosynthetic genes during post-harvest ripening of Monthong and Chanee, the HMM profile of ACS and ACO (PF00155 from the Pfam protein database (http://pfam.xfam.org/)) was used as a query to search against our in-house transcriptome database and the Musang King genome (i.e., *ACS* (XM_022901720.1) and *ACO* (XM_022903266.1). To perform qRT-PCR, primers were designed as mentioned previously (see primers in Supplementary Table S3).

### Transient expression of *DzDof2*.*2* in *N*. *benthamiana* leaf

The full-length cDNA of *DzDof2*.*2* was amplified from the cDNA using Phusion Hot Start II High-Fidelity DNA Polymerase (Thermo Fisher Scientific, USA) (primers are listed in Supplementary Table S4). Then, the PCR amplicon was introduced into pDONR207 vector using Gateway® BP Clonase® II (Thermo Fisher Scientific, USA). The putative *DzDof2*.*2* from pDONR207-*DzDof2*.*2* was inserted in to the pGWB2 expression vector using Gateway® LR Clonase® II (Thermo Fisher Scientific, USA) to produce pGWB2-*DzDof2*.*2*. The resulting pGWB2-*DzDof2*.*2* as well as pGWB2-*GFP* (control) were individually inserted into the *Agrobacterium tumefaciens* strain GV3101 by electroporation. The positive *A*. *tumefaciens* colony containing each construct was grown in 25 mL of LB broth containing 25 mg L^−1^ gentamycin and 25 mg L^−1^ rifampicin at 30 °C with shaking at 250 rpm overnight. Cells were harvested by centrifugation at 4,000 × g for 10 min and resuspended in MM buffer (10 mM MES and 10 mM MgCl_2_, pH 5.6) to an optical density of 0.6 (OD_600_). For agroinfiltration, the agrobacterium solution containing either pGWB2-*DzDof2*.*2* or pGWB2-*GFP* (control) was mixed with the agrobacterium solution harbouring the gene encoding the silencing inhibitor protein p19 at a ratio of 1:1. Thereafter, acetosyringone was added (100 mg L^−1^) and the mixed culture solution was incubated at room temperature for 3 h under dark conditions. Then, the solution was used to infiltrate the abaxial surface of three individual leaves per plant using a needleless 1-mL syringe. For each construct, three 4-week-old plants were used. After three days, the infiltrated leaves were collected, immediately frozen in liquid nitrogen and ground into a fine powder which was then used for RNA isolation and cDNA synthesis. The cDNA was further used as the template for qRT-PCR analysis. In order to design primers for *N*. *benthamiana* auxin biosynthetic genes, the *Nicotiana tabacum* genome (i.e., *TAA1* (XM_016657015.1) and *YUCCA3* (XM_016640518.1)) were used to blast against the genome sequence of *N*. *benthamiana* (SGN *Nicotiana benthamiana* ftp site; https://solgenomics.net/organism/Nicotiana_benthamiana/genome)^51^. Primers are listed in Supplementary Table S4. The 2000-bp upstream promoter regions of these genes were scanned for the Dof domain (AAAG/CTTT) using the online tool PLACE (http://www.dna.affrc.go.jp/)^36^.

### Statistical analyses

All experiments were conducted using three biological replicates. Statistical comparisons of the mean values were conducted using one-way ANOVAs, followed by Duncan’s multiple range test or Student’s *t*-test at the 0.05 significance level using the statistical package for social sciences (SPSS) software, version 20. All data were represented as means ± standard deviations (SD).

## Supplementary information

Supplementary Information
