# Supplementary material for: Genome-wide analysis of the Dof gene family in durian reveals fruit ripening-associated and cultivar-dependent Dof transcription factors
Source: Sci Rep. 2019 Aug 20;9:12109. doi: 10.1038/s41598-019-48601-7 (PMC6702166; doi:10.1038/s41598-019-48601-7)
Supplement: Supplementary file 1 — Supplementary Information [file 41598_2019_48601_MOESM1_ESM.pdf]

Genome-Wide Analysis of the *Dof* Gene Family in Durian Reveals Fruit Ripening-Associated and Cultivar-Dependent Dof Transcription Factors

Gholamreza Khaksar[1], Wassakarn Sangchay[1], Pinnapat Pinsorn[1], Lalida Sangpong[1], Supaart Sirikantaramas[1,2,*]

[1]Molecular Crop Research Unit, Department of Biochemistry, Faculty of Science, Chulalongkorn University, 254 Phayathai Road, Bangkok 10330, Thailand

[2]Omics Sciences and Bioinformatics Center, Chulalongkorn University, 254 Phayathai Road, Bangkok 10330, Thailand

[*]Corresponding author. E-mail: supaart.s@chula.ac.th

Supplementary material for this submission is available as follows:

Supplementary Table S1

Supplementary Table S2

Supplementary Table S3

Supplementary Table S4

Supplementary Figure S1

Supplementary Figure S2

Supplementary Figure S3

Supplementary Figure S4

Supplementary Figure S5

Supplementary Figure S6

Supplementary Figure S7

Supplementary Figure S8

Supplementary Table S1. Sequence similarities among DzDof sequences. Numbers indicate percent homologies at the amino acid level. The protein sequences of 24 DzDofs were used as input queries to find sequence similarities using the web-based tool Clustal Omega (https://www.ebi.ac.uk/Tools/msa/clustalo/), using its default parameters. The highest and lowest similarities are highlighted in yellow.

		DzDof 1.1	DzDof 3.7	DzDof 5.7	DzDof 2.2	DzDof 3.6	DzDof 2.4	DzDof 5.1	DzcDof 2	DzcDof 3	DzcDof 1	DzDof 1.5	DzDof 1.4	DzDof 1.2	DzDof 3.5	DzDof 4.5	DzDof 5.3	DzDof 5.4	DzDof 3.1	DzDof 1.6	DzDof 3.4	DzDof 2.5	DzDof 4.6	DzDof 2.1	DzDof 5.6
1	DzDof1.1	100																							
2	DzDof3.7	85.58	100																						
3	DzDof5.7	38.26	37.79	100																					
4	DzDof2.2	34.71	34.85	34.15	100																				
5	DzDof3.6	31.56	30.15	32.06	55.48	100																			
6	DzDof2.4	31.56	30.15	34.22	56.41	56.97	100																		
7	DzDof5.1	31.68	29.50	33.46	53.23	56.13	89.85	100																	
8	DzcDof2	26.64	24.61	28.23	25.48	23.76	25.18	24.91	100																
9	DzcDof3	27.82	26.53	28.86	28.63	26.59	25.84	25.19	58.63	100															
10	DzcDof1	26.61	24.80	24.72	25.46	23.94	25.44	24.74	46.79	43.42	100														
11	DzDof1.5	34.51	33.33	31.76	33.81	33.56	31.97	31.29	40.26	39.60	40.70	100													
12	DzDof1.4	29.91	28.91	29.90	31.63	30.59	29.82	29.86	25.35	28.14	24.56	35.83	100												
13	DzDof1.2	23.66	25.79	27.63	27.23	28.32	28.63	27.88	25.50	25.51	22.82	31.51	29.55	100											
14	DzDof3.5	28.99	29.76	27.57	31.05	28.28	35.35	34.36	27.03	28.96	26.32	33.59	34.34	38.02	100										
15	DzDof4.5	33.66	34.65	30.43	33.95	31.53	32.74	30.80	30.20	29.15	25.00	34.75	32.09	27.97	29.91	100									
16	DzDof5.3	32.56	32.55	31.80	35.87	32.33	34.62	33.62	30.81	28.37	25.22	33.33	31.25	28.74	29.57	87.86	100								
17	DzDof5.4	33.33	33.33	31.52	34.95	35.57	31.47	30.85	27.32	27.80	23.01	38.79	33.78	29.47	29.41	32.79	33.33	100							
18	DzDof3.1	36.09	35.93	36.69	36.14	33.92	35.88	35.43	32.05	31.61	28.57	43.30	33.15	32.78	31.07	33.80	34.40	35.68	100						
19	DzDof1.6	32.75	35.12	31.32	30.90	30.60	29.28	30.11	31.55	31.33	27.23	42.31	29.28	28.72	31.07	31.28	29.22	32.73	41.01	100					
20	DzDof3.4	32.00	32.56	32.18	36.47	32.95	34.10	33.15	31.93	29.52	25.82	39.22	32.39	31.15	30.86	28.64	26.76	36.57	43.93	47.16	100				
21	DzDof2.5	35.96	32.84	32.98	36.87	34.74	35.79	35.23	30.69	32.31	28.43	33.06	29.36	31.60	33.17	35.24	34.55	32.86	37.10	32.80	33.51	100			
22	DzDof4.6	32.68	31.53	34.21	40.46	38.38	39.46	37.97	29.95	30.53	29.23	32.00	30.52	30.19	34.15	32.85	32.72	33.65	38.04	33.33	33.70	64.93	100		
23	DzDof2.1	28.44	30.62	35.61	33.67	33.17	32.18	31.22	32.62	32.61	26.44	38.53	34.50	35.27	31.86	35.24	34.04	35.02	39.18	36.41	31.77	37.77	39.30	100	
24	DzDof5.6	26.58	28.31	31.12	31.58	30.05	30.99	32.41	25.12	26.24	25.70	32.17	30.00	30.63	32.84	32.75	33.05	32.46	33.99	30.50	30.15	34.90	36.00	38.85	100

Supplementary Table S2. List of primers for *DzDofs* used in this study.

Gene name	Corresponding gene ID	Forward primer (5'-3')	Reverse primer (5'-3')
DzcDof1	XM_022890912.1	CCCGGAAATGTCACCGTCTG	AGTGTTCAGACCAGAAAACAACAC
DzcDof2	XM_022884508.1	CCAACTTCGTTAGCTTAGCCAC	ACAGGAACTCTTCTTCTGTTCTCC
DzcDof3	XM_022882662.1	ATTGGAGCTGCTGAAAAGGGC	GGCACCATCAGCCACAATTAAC
DzDof1.1	XM_022885861.1	CTGATCTTCCAGCAACTAATGGTG	AAGGCATATACAGCTAGCCAACA
DzDof1.2	XM_022897927.1	TTCCCACATGAATTGGCTAGT	TAGAACCAACCCAGTAACGGT
DzDof1.4	XM_022864763.1	GTCTCTACCGATCTCAACATGCC	GAACACCCAAATCAAGAAAAGAGC
DzDof1.5	XM_022921573.1	CGAGACTTTGGAGCAAAACCC	GACCCATTGACATCACAGGA
DzDof1.6	XM_022916439.1	TGCAGCAGAACACTCGTGA	GGTGGTTTAATTGGCAACGCC
DzDof2.1	XM_022896728.1	GCTGACCAATAAGGCAGTTCC	CCGAAGTCCTGCAACTAACCA
DzDof2.2	XM_022874863.1	GCAGACTAAACAAAGATTGGCAG	CACACACAACCGTAGAACAAATC
DzDof2.4	XM_022888412.1	TGTCAAGGCAATATTTGGGGG	ATGATTGCTAGTGGATGAAGAGC
DzDof2.5	XM_022903860.1	TGGGTTTGGTTTCAAAAGGCTG	CAACACTGACAGAAGCAGTCC
DzDof3.1	XM_022865170.1	TCCGGGATTAGGAGAGAGCAAT	GAAAACTTGAAGAACCTGGAGTG
DzDof3.4	XM_022918231.1	GGTGGCGATTACTTTTCTTGGT	GGAACATGGAAAACACAATGAACC
DzDof3.5	XM_022889354.1	CTGTGGTCGAATACTCACTCCT	TACCAAACAATAGATTTGGATCGTG
DzDof3.6	XM_022908803.1	CGAATGAATTGCTTGTCACTCCA	TCATGTCAGATCAAACTCTCTGC
DzDof3.7	XM_022895569.1	TGTGGCTGTAGGGGTAGCA	CCATCCTGGTACACTTTCACAT
DzDof4.5	XM_022921611.1	ATGTGGGTGTTCGTTCCTGT	CGCACCAACAAAGCAGAAGT
DzDof4.6	XM_022877869.1	TTGGGGTAAAAATGTAGGAGAGGAG	TGACGCATATGGTTGCTCTTTC
DzDof5.1	XM_022891906.1	AACCTCCAGACACCTTATCCA	GCGCAATGCATATCATCAGTAGG
DzDof5.3	XM_022868588.1	TTTCTTCAGCCACTGCTGTACT	GCTTTTTGCATACACAAGGCAA
DzDof5.4	XM_022920120.1	TGGCAAGGGAGTGGAGATCA	ACGGGATATAGAGGGTAGGATG
DzDof5.6	XM_022881477.1	AAGGGGAAACTGTGTGGTGAAT	CTTTTGCCCAAGACACCCAATC
DzDof5.7	XM_022885261.1	GGTAGACCCTAAAAGGAAGATATGG	CAAGGTCGACATGTTGATATCTGA

Supplementary Table S3. List of primers used for expression analysis of auxin and ethylene biosynthetic genes in durian pulp.

Gene	Forward primer (5'-3')	Reverse primer (5'-3')
Auxin biosynthetic genes		
DzTAA1	TTGAATATGGACAGCGCCTCA	AAGTTGCAGTAGTCTGGCGA
DzYUCCA4	TAACCCTCCAAAACCGCAGAA	TTCCCAAAGTACCCGACCAAG
Ethylene biosynthetic genes		
ACC synthase (*DzACS*)	CAGAAATCATTGGCCTGGTAG	CTGAGTTGGAGCTGAAATGG
ACC oxidase (*DzACO*)	TGGAGAAAGAAGCAGAGGAG	AAGTTGTTTCCCTGGCTTTC
Elongation factor 1 alpha (DzEF-1α)	GAAACCTTCTCTGCGTACC	CTCCACACTCTTGATGACAC

Supplementary Table S4. List of primers used for transient expression of *DzDof2.2* in *Nicotiana benthamiana* and expression analysis of auxin biosynthetic genes in *N. benthamiana leaves*

Gene	Forward primer (5'-3')	Reverse primer (5'-3')
For gene cloning		
DzDof2.2	AAAAAGCAGGCTATGGTTTTCTCATCTGTTCCAG	AGAAAGCTGGGTCTATAAGAGATTATTAGTACTAGAAGAGTTGAGACC
For expression analysis of auxin biosynthetic genes		
NbTAA1	ATACTGGCGAAAGATGGGCAAC	CTGGCTTGATCCTGTCCCAAC
NbYUCCA3	AAGCTGATAGTCTGTAATGAGGGG	TTGGGATTTCTCTGGAGAGGG
Elongation factor 1 alpha (NbEF-1α)	TGAGGGACATGCGTCAAACT	GGAGCGGATACCAGTCATACA

Underlined sequences represent bases of the *att*B1 or *att*B2 site on the 5' end of each primer (for BP reaction).

Supplementary Fig. S1. Exon-intron structures of *DzDofs*. Green bars indicate exons. No introns were found for durian Dofs. DzcDof is durian cyclic Dof.

Supplementary Fig. S2. Multilevel consensus sequences identified by MEME. Protein sequences of DzDofs were used to identify conserved motifs among durian DzDof proteins. Ten conserved motifs were identified. Motif 1 represents the conserved Dof domain always observed at the N-terminus.

Supplementary Fig. S3. Schematic distribution of conserved motifs in durian DzDofs. Ten conserved motifs, identified by MEME, are represented in different colours. Motif 1 represents the conserved Dof domain. Several subgroups were distinguished based on their motif distributions, which was consistent with the phylogenetic analysis.

Supplementary Fig. S4. Tissue-specific expression profiles of *DzDofs*. The Illumina RNA-seq data were used to analyse the expression levels of *DzDofs* in different tissues, including root, stem, leaf, and pulp. The higher expression for each gene was presented in red; otherwise, blue was used. Data were sum normalized, log transformed and autoscaled.

Supplementary Fig. S5. Comparison of the expression levels of ten potential cultivar-dependent *DzDofs* between the Monthong and Chanee cultivars. Expression levels of ten potential cultivar-dependent *DzDofs* during ripening were compared at three different stages (unripe, midripe, and ripe) between the Monthong and Chanee cultivars. For each *DzDof*, a comparison was made between the two cultivars at each stage. Three independent biological replicates were used. Three technical replicates were also performed for each biological replicate. An asterisk (∗) above the bars indicates a significant difference between the two cultivars (Student's *t*-test, $P < 0.05$).

Supplementary Fig. S6. Expression levels of ethylene biosynthetic genes in durian pulp. The expression levels of ACC synthase (*DzACS*) and ACC oxidase (*DzACO*) were measured at three different stages (unripe, midripe, and ripe) during post-harvest ripening of Monthong and Chanee cultivars. Comparisons were made between the two cultivars at each stage. Three independent biological replicates were used. Three technical replicates were also performed for each biological replicate. An asterisk (∗) above the bars indicates a significant difference between cultivars (Student's *t*-test, $P < 0.05$). Comparisons were also made for one cultivar at different stages. Bars with different letters (lower case for Monthong and upper case for Chanee) above them differed significantly according to Duncan's multiple range test ($P < 0.05$).

Supplementary Fig. S7. Nucleotide sequences of promoter regions of candidate auxin biosynthetic genes from durian (*DzTAA1* and *DzYUCCA4*). Dof binding sites (AAAG and CTTT) are highlighted in yellow. The translational start site (ATG) is underlined.

Supplementary Fig. S8. Nucleotide sequences of promoter regions of candidate auxin biosynthetic genes from *N. benthamiana* (*NbTAA1* and *NbYUCCA3*). Dof binding sites (AAAG and CTTT) are highlighted in yellow. The translational start site (ATG) is underlined.

Supplementary Fig. S1

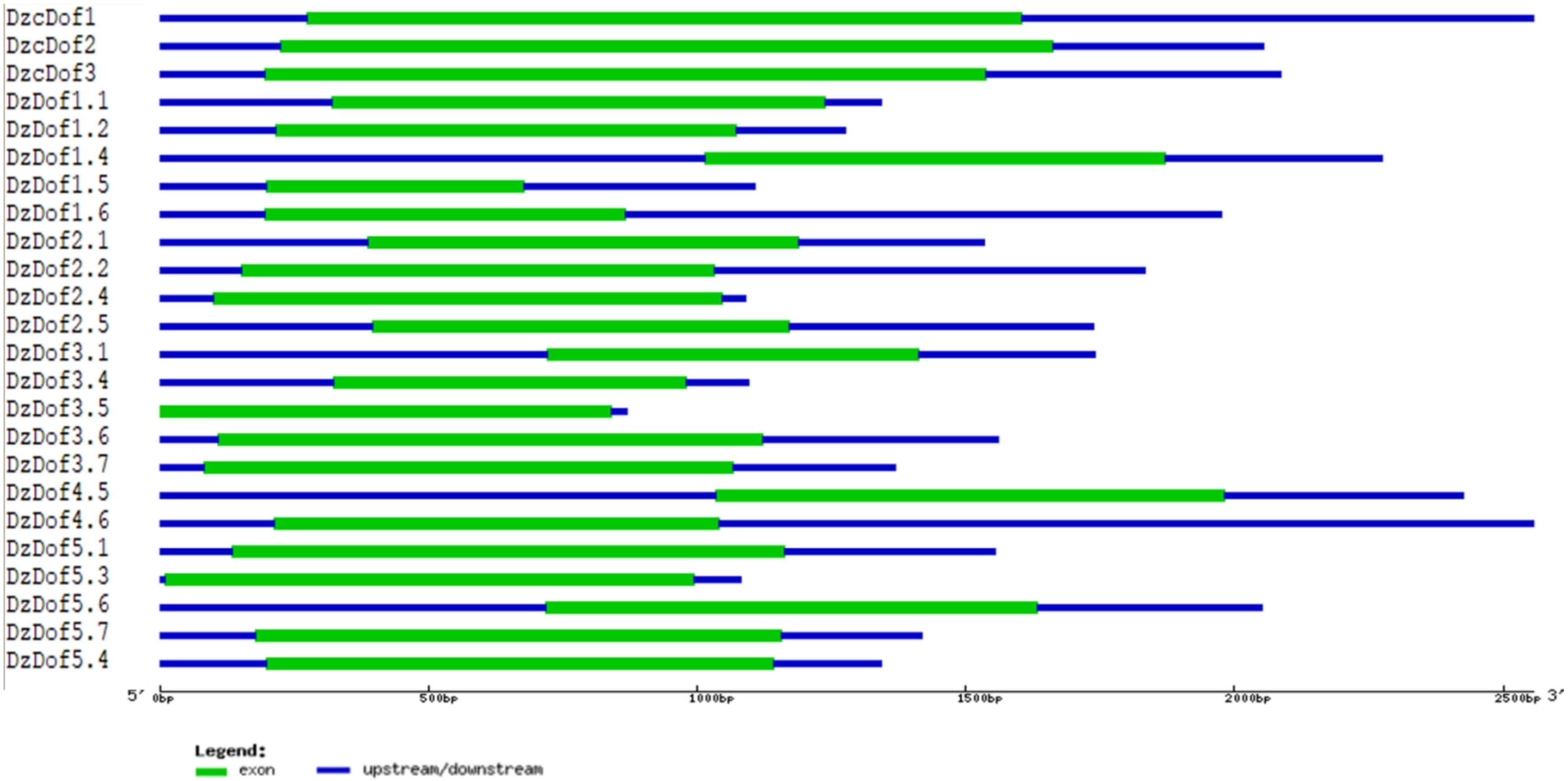

Supplementary Fig. S2

	Logo	Amino acid sequence	Length (aa)
1.	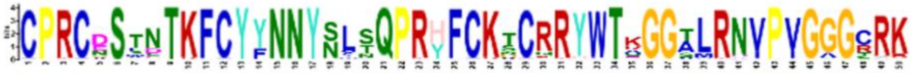	CPRCDSTNTKFCYYNNYSLSQPRHFCKTCRRYWTKGGTLRNVPVGGGCRK	50
2.	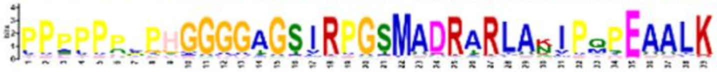	PPPPPPLPHGGGGAGSIRPGSMADRARLAKIPQPEAALK	39
3.	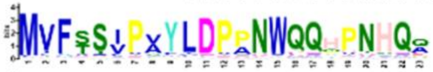	MVFSSIPVYLDPPNWQQHPNHQQ	23
4.	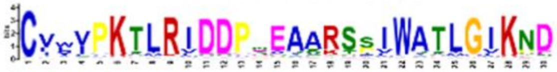	CVWVPKTLRIDDPNEAARSSIWATLGIKND	30
5.	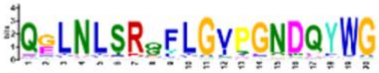	QGLNLSRQFLGVPGNDQYWG	20
6.	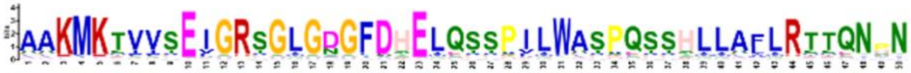	AAKMKTVVSEIGRSGLGDGFDHELQSSPILWASPQSSHLLAFLRTTQNPN	50
7.	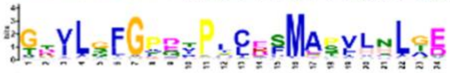	GTVLGFGPDTPLCESMAPVLNLGE	24
8.	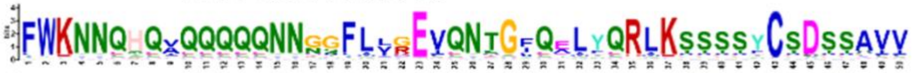	FWKNNQHQVQQQQQNNGGFLLGEVQNTGFQELYQRLKSSSSYCSDSSAVV	50
9.	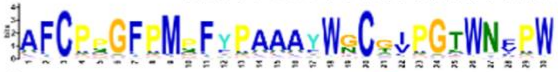	AFCPPGFPMPFYPAAAYWGCGIPGTWNFPW	30
10.	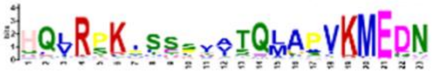	HQLRPKISSSVATQLAPVKMEDN	23

Supplementary Fig. S3

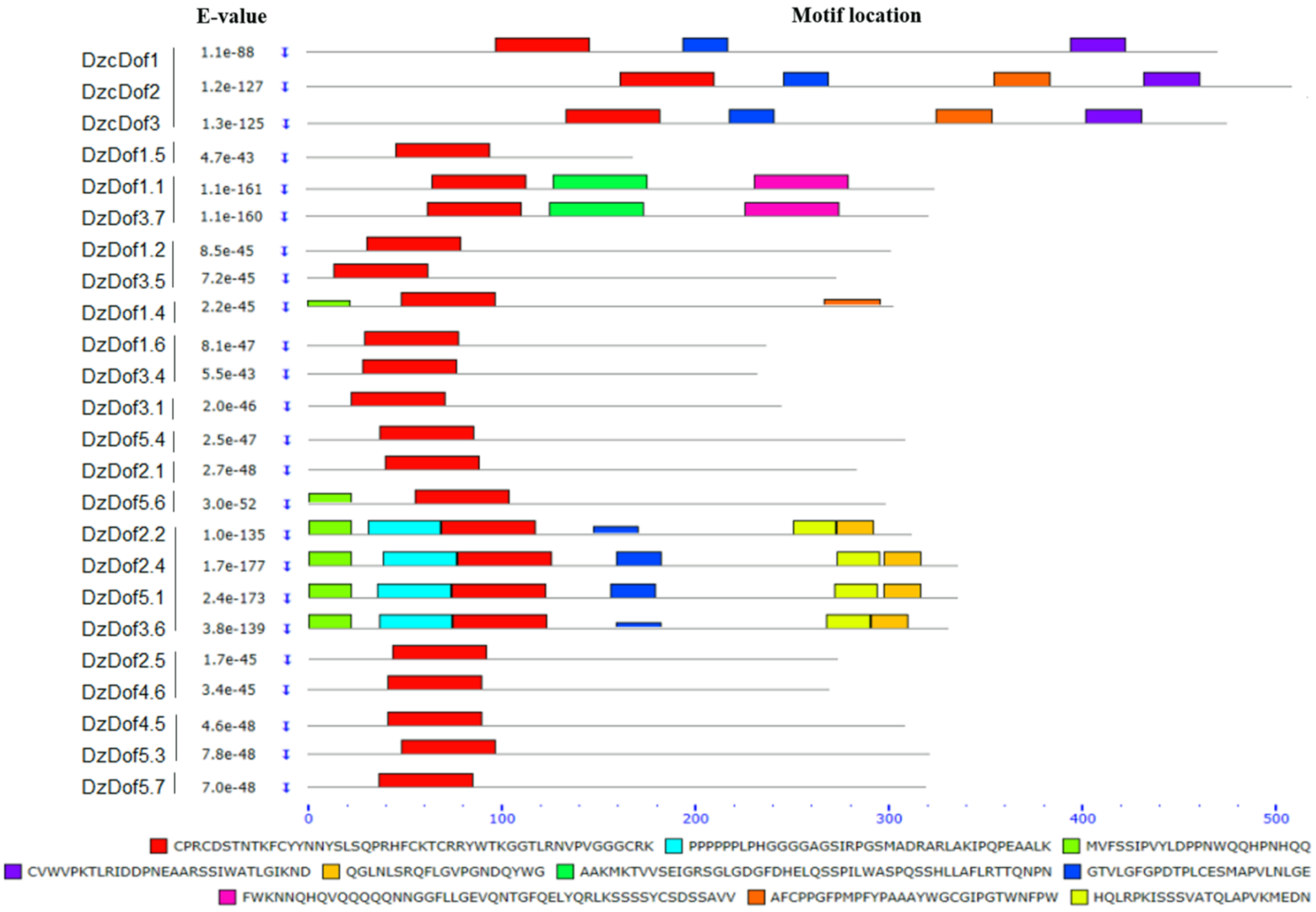

Supplementary Fig. S4

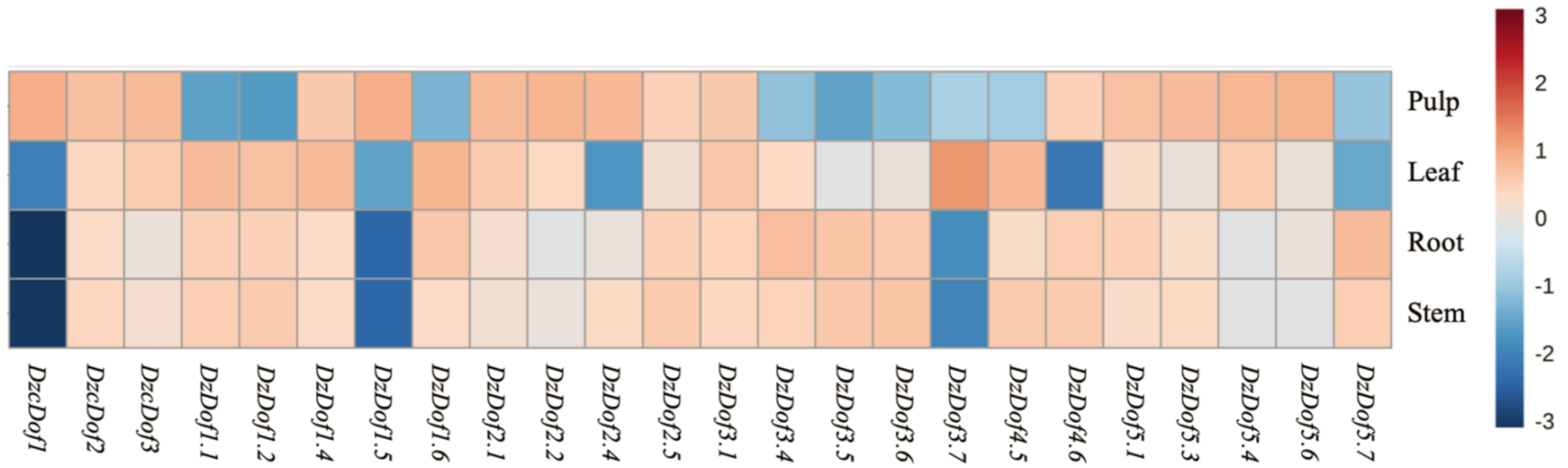

Supplementary Fig. S5

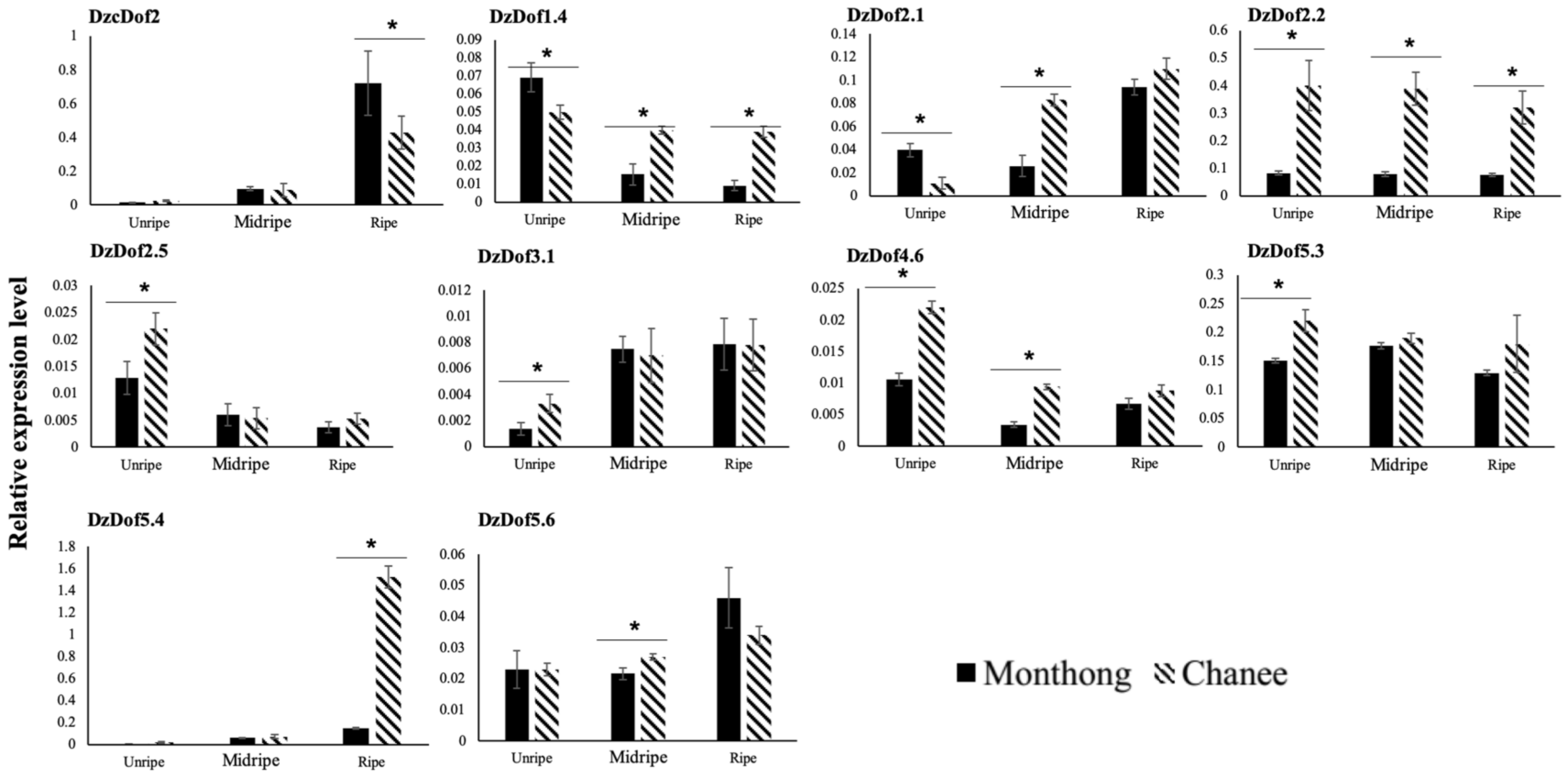

Supplementary Fig. S6

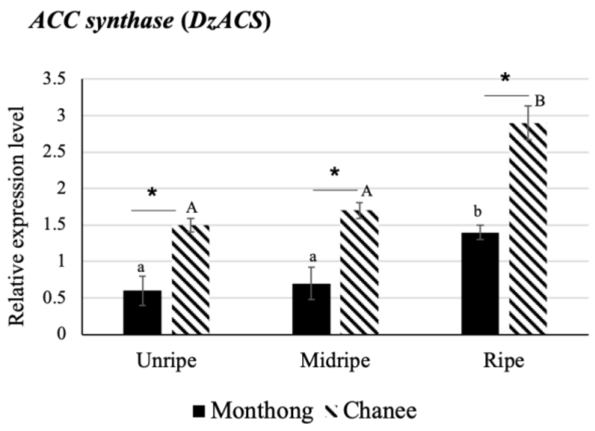

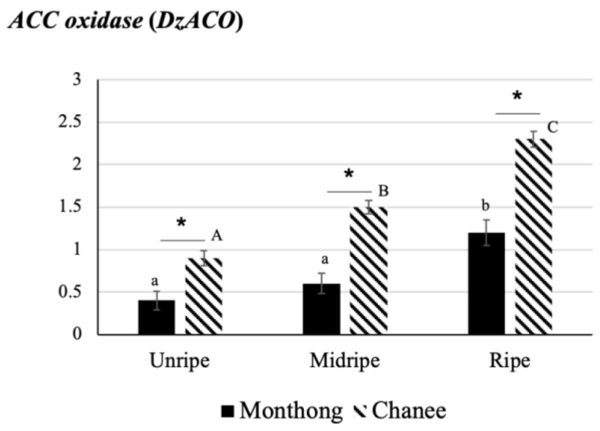

Supplementary Fig. S7

Promoter region of *DzTAA1*

ATAATGTTTGATTAGTTGTTTGAAAGATACTATGAGAACACATATAACCATGAATTTGCTATTCAAAACC
CATGCGTTAAGAATCAAAAATAAATTTTTTAAAGTTAATTGATTGGTGGAAATACTTATTTGTGACAAAC
CATTTTAGTTTCCATAAAAAAGTTCTTTAAAGCAAATAATGGTGTTCTCATCAAGTGTATAAAAAACATT
TTAAAAGTATGTTTTTCACTAATTCTGTTCGAAAACTTTTGAAGTTTTGTCGTATATTTCGTAAAAAAAT
TATAAGTTTTATGTAAATGTTCTACACCACTCAAACTCATTTTTATTTCTTATTAATTATTATTTTTATT
TTTCATATTTCATTCCCTAAATTTTCTAACGGTTACTATAATAAACCAAGTCTAGTTCTAGAGCATTTAG
TTATCAAAATTCAATATGAAACAATTTTAAATATATAAAAAGAAAAACCAATTATAGAAATTCCAGTTTT
AATTTACTGGAGAACGTGATTTGGTTAGGTTTAGCATATATAGTAAGTATTCCTTTAGATACAGTCATTG
TGATTCCGAGATTTTCTGCCTTTCAAGGACTTTTTTGTTGGGTTCCCTTTAAACAGTATCCGACAATTTT
GTGGGAAACCTAAATTAAGAGCTTTTTCCCAAAGTAAGTTGCTTTTTAATCTTATTTCTTTAGTAAGGGT
ATTCTTAAATTTTATTTCAAAATAAAGTTATTTTGAGTCAATTAACCTACATGTCAGCATTTTATTCCAC
CTATCGTTTTCTTTTGAATTTACGTTTTGTTTCTCTCTTGTACGTTTTTAACACATACATTCAATTTTTT
TTTTCACAAAACCATTCTCCAAAATCAATATTGCTTTAGATCTCAATTATGAGACAAATATATTATTACA
AAAAAAAAAAGAGTAAGCTTTGATTTGCATTCATTAGAGAATTTTTGTTTATGGCAATTATTCATAAG
AAAATTAAAAAAAAAAAATTCTATTGCAATTTAAAATCAGACAAAAACATAACAATAAGTTCCTTGCTGA
AGGAGAAGACATGTGTGTCCATATGTTTGATTTCCATATTGGATATGGGTTTGGTTGCAACAAGGATCCA
TTATAATGTTGTCCACTTTGTACGTTTCTCATCTAAAGGGAAACCTAAGCACCAGGAGAAGATGATTTCA
AAATGACAATTCTTTTTTGTAAGATTTTAAAATCCACTACTTCAAATTAGGGAGAGATGAAAGATTAAAG
AATTGGGGAAAAAAAAGGTTATATCATCAAAATTAATAAGATTTTTTTTTTTTAAAATTCTGGAGAGGCA
AATAGAATCCAAGTTTGATGATTTCATTTTTACTACTTCTCGAGTTTTGTAGCTTTTTTTTTTTACTGTC
ATTCAAGTTATGCTTATTCATGTCGATATGCAATTATTTATATGTACTTTGATATTAATTTTGGAGTAAG
ATAAATAGAATGAGATATAAAGATTATTTTGACAAAGATTATACTTTTTCATGTGGGTATGTAATCATTG
GTATAAATTTTGGAATTGATTTTAGAATAAGAAAATTAGAATAAGACAGAGAAATATAAATTTTGAGATT
GATTTTAGTGTAAAAAAATAGAATGAGATAGAGATTATTTTGATAAAGATAAGAATGTGATTGTTATAAG
TAAGTAGAAATAAATTATTTTTATTTTATTAATATGAGATATTGACATGGAAAAAGTACGCTGATTTGTA
GTCCAAATAATGTAGAGTACCTTTGTTTAGGGATTAAGTTTAAAAGTACTCTGGTCAGGGAAATAAAGTT
TCGAAGTGTCGTAGTTTGGGAAAAAACTCTGAGTTAGGGTTCATGAACCTTCCAATCTACAGCATTTCTT
CCCCCCTATAATACCCATCTCCACGTTCCACTATCCTTCCCTGAGAGAGGTCCCTTTATTATATATACAG
GCAGAAAAGTTATTTTACAATCACTTTTGGAGAAGCCAGGATG

Promoter region of *DzYUCCA4*

ATTTGAATTCGTTTAAATATTCGTTTAAGTTTAGTATCATTATCAAAATTTAAATCTATTTAAACAAGTA
ATATTCTTTTAAATATCTATTTATTTAAAATTTTTTGTCAAAATTAATATAAAATAAACGAGAATTAAAA
TTATATTTTAAATTCACTATTAATTAATAATAAATAAAATTATTTATTTAGGTTCAAAATTTTTCACAAA
CATAAAATTTAAATATTTATTATTATTTATTAAAATTTAAACGAATTCAAATGTTATATATTCAATAAAT
ACCATCCATATTTTAATTTTCACGAATGATAAATTTTTTATCTAAATTCACTAATTATTAATTTATAATT
ATTTAAATTTAACATAAACACGAATATTCATTCATTTGCTATTCATTCTCATATTTAATTGTGACCAAAT
AAATTATATCTCAACTACCAATCTAAACTAAATCAACTAGAAGCTATATATGCATGCTTTAGCAAGAATA
CATTTTAAAAAGTTAATGGTATCTTCGGACGACCTTAGTAATTAAAAAAAAAAATCCTATGCTCAAGCAA
CCAATCTCAATAGCTCGTACTAATAATGAAAGGCACCAAGACCTAACCTGGTCAAAAGTTATCGCAAGAA
AGTCGGCCTATGTTTTATCTAATTAACAAGATGTTTTTAGGTTCTATAATAATAAAATAATACTATAGGT
TTACAGAGCACTTTTCGTGCCTTTTGCTTTCTTCGCATGCTTTTGCGCTTGCATACTACTTACTAGAATG
CAATACAATACACACACAAAAATAAAAACTTTATAAATATTATATTTAAATTATAATGATTATGATATAA
ATTAATTAAAATAAAAGTGAAATTAATACTTTATTAAAAAATATACACATAAGAAAAATTTTCAATGTGA
TTTATTTGCATGGTCATTATAATATAAGAAAGAGTTTGAATTTTATTAAATTTTCAATGTAATGTAATTG
AATTTAATGTATCATTAGTTTAATTTAAATATTAATTTAAATAAAATTTATTTAATATAAAACTTTTAAT
CATAATTATAATAATAATTATTATTTTTAAATTTTATCACAATTAATTTTATCATTTAAATTTACAAGAC
AAATTTTCACGAAAGCCAACAGATGTCCTGTCAACAAGGTTTCTTCTGATCGATCTTTTCAAAAGAATAA
TTAAACAGTCAAAATTTTAACTTTAATCTTTAAAATTAAAATTAATTAACCTTTTACTTCTATTTTAATT
TTAATAATTAAGATTTAAATTTTCACGGCTCAAGTAAAAAAATGTTTCTTCTTTATGTTGTTAAACAAAA
AGTAAAATACGTTCTATTTATGAAATTATTTTGTACAAAGTTAGCAAACTTACATCATCATAAGATGCAA
ACCCCTCGTACAAAATTGGTACATTTACAATATTTTAAAAGAGGGGTTAAAAAAAAAGGTCACCCTGGGT
AAATTCCGATGAAATATTTTTTTTTACTTTTTCACCTTTTGCTTCTTGAATCACGTTTCCTTGAGCAGGA
TAACATGAGAATTACAACTGTTGTTGTTACAATCTTTGATTGTCCTCCATTGTTCAGCAATATCACCAGC
AATCTTAAAGGCGTCAGAAGCCGTTCCTAGGAGCCCTCTTCTTGTAAACCCAACTGTGTACAATCCCTTC
TCTGCTTTCCATCCGTTGGGAAAAGGCGTTTTTGGCATACCATCTTTGGTGAAAACATCACACCCCTATA
TTTCCATTAAGGGCAAAAAAAACAATGTTACAACGACTAATAAATACAACTGCTTCAAAAGATCAATAAT
TTGTACAAATGAATTGATGAATAAGAGTGGAAAAAGCTGTGAAAATCATATCAAACTGAACCCATTTTCT
GAAATTTCTTCTAGGAAAAGGCAATCGAGGGAATTTTCTAGGTTTTTTTTTTTTTTTTTGGTTTAAGGC
AAATGAATCCTCAAAAATACCCATTATTTAATGATGATTAATG

Supplementary Fig. S8

Promoter region of *NbTAA1*

AGTTTACACGTAGGTTGGACGTTTGTTTAGTCTTTACCGAGACGAGGGAGGTTAGGGTGGGTACGGGGGT
TTAGTAATAGAGCTTAGGAGTTACATTTCGGGAGCCTTAGGATTTAGTTTTAGTGTCAATGCAAATGAGT
TTAGCCAGTTTGCTGTTTCCAATCTGACATGCTGATGCAGTAAACTCGGGACTTTGTAAGTAAAAACTTG
ATAAGAAGAAATACTAAAAGGAAACACAGAGACAATCTGTGTATTTGATGTTGTTTAGTAAGTCTTGCCA
AAGAAAATAAGTATATTAGCTCTCTTGCTTAGAATTGCATAAACTTGTGCAGATGGAGAGACCTTATGTT
TTGCCTGGAGTTGTGGTAGAGCATTGCTTTAGCACTGGCCTTCTACACATGCATTGTCATGGGCAGCACA
TGTATCATCATTCAATTGCACATGCCATTCTTATTTTTGCAGAATCAAACCATCCGACCACAACTCACTA
TGAAAGTCTGATACAAATATTTTTATACTTTTTTTTCAGTTATTGAATTTGAGGGGAGCCTTGGCACAAC
AGTAATGCATGCTGCTTCCCTGGTATCTAGTAGGTCACTCGTTTGAGACATAGAAATAACTTACATTTGC
ATAAATGCAAGAGAAACATAGCATACAGTATACACGATCGTTCTCTCCTGCTCTAGTTGTAGAGCACTTG
GTATACTGAGTAAGCCCGATTTTCAGATGGATATTGAGTTTGTTTTTTTGAAGAAGATAAAAATGATGAT
GGCCTAACGGTCTTAATGAAATCATCGGAAATAGGATAACAATTCTAGATAAAGACAAAATCATATGCTT
AATGCATGGTCAGCAAACTTACTTAGTGTATGTGTTATTAGATATGGTAATAGATAATTGATAAAATAGT
TAAGGTTCGCGTAAGTTACTTGAATATCACGCTATCTAAAAAGGACTTTCTTAATAAAAAAAAAAAAAAT
TAGTAGTACAACATTTCTTTTCTCTTTTTCTGTACATGGCCCTGCAAAGTTACTTAGTATATGTACTGTT
GAGAGGTAACAAATAACTGATAAAATAGTAGAGGTTCGCGTAAGTTACTCGAACACCAGGCTAATTAAAA
GACACTATTTTGTGATCTAAATTTATACGACTGACTATTCCAACATACACAATTAAAGCGCCGACCAAGA
GCCAGCAACATAACGACCCAACATCCATACAGAAACCAAAAAGATTCCTAAAGAGAGAACTCAGTTAAGT
TTCATCTTATTTTATAGAGTTCGATTCTCTTTGATACCAACAGTCAAACTTACATCACATGCAGTGATGC
AGTTCTTCTAATTTAATGAAAACACTTAAAGAGACAAAAAAAATAAAAAGAACTGACCATATTATTTTGT
TTTTCATTTTTATATTTTACCGTTGTCTCTTCAATTTTTTCCCTATCTGTTTTTTCTTTTATTATAAAAA
ATACAAGTTAGTTGTTGACTTAGTAGTATCTTTGATCAAAATGGATAAACACAAAAATTTTTCCCTTAAA
TCAATCTAGATAAACACAAAAAATTTCCCATATGTGTCAAAAATACAATTTCCTTTGTTTAGATCAAGAG
ATTTAATCTCCTTACTATACGGACAAAATTGCAAGACTCTATCTTATCTGCTTTCAACTGTTTTTTACCA
TTCAAAGTATTCTCTCGAAACAGCTAAAATGCAAAAATCAAGATTAGAAAAGACATAATTACACTTATTA
GGGAATCCATCTAAGATTCTGCCTATTAATTAGAAGGCTTTTTATGGAGAATTAGTAAGAGAATCCATTT
AAAATTCTATCTATTAATTAAGAGACCTTTTGTGCATTAGCCTTTGCCTCCTCCCCTATATATAGTGCAG
CATGCAAGACATGGATTAGTACAAAAATCTGTCTTCTCCAATTATAAACATATCCTAAATAGCAATAGCT
TATTTTTTGATTGATCAAAGGTTTTAGAAAGAAAAGAAGAATG

Promoter region of *NbYUCCA3*

CAAGTCATTTTAATTAAACTAACGCACTCTCAAAATTCAGTATCATATATTCATATGATTGAATGGCCAA
AATAATCAACATATTATTACTCTACCTGAGATGAAACAGAGTGAATCTCACTTCAAGCGTAGTGCACAGA
ATACAAGTTATTTGATCACTTGGTTAAACCAAAATTTGCAAGTTGAAGCTTAGTAGATTGCTTGGCAAAA
CCAAATTTTATTTGCAAAAGATTTGATCAACCTAGAGGAACTCAAACTTGATATGTGCAATTGACTAAGT
TGATATGATGAGATCTCACTACCATCATAATCCCTCTCATTCTTCATCATATTATAGTTTGAACTTTCTT
CTCTTTTCCAAGAAACTTTGGAGCAATTATAAAATTTTCTCTGTGTAACCTATAGATCACATGTTCGATC
CGTAAAATCAGTCATTAATGTTTGCATTAGGGTAGGTTGTCTACATTACACCCCTTGAGCTGCAGCCCTT
CTCCGGACCTTGCGTGAACATGGGATGACTTGTGCACCGGGCTGCCCTTTTTCGCCCTCATTTCCATCAC
TAACTACACTAATTACAATAAGAAACTTGGATGGTGTAATATGGGATTCAAAATCTTGCTTGGAAAACTT
GTGCTCTCGGAGTATGTAGACAACTCAATATGTTTTATAAAAAGTTTCCTACTTAAGTTAAATTTAGAAG
ATATTGAGAAATAGCTGCACCTCATGTTTTTCGTTGGTTTTGTTTGTCTTTTGAAGGGTTTGCTTATGGG
GTTTCATGCTCAATGTTGCTTTTAGGTGGAAAAGAATGGAAGAGAGAGAACACACATTCAAAAAGAAAAT
GAAATTTGGATTTAGTGAAAGGAAAATCATTTATTTTTTCTTCTTGAGTTGGCTTTGGGAATAAAATGGA
TAGAGTAGAAAGAGAAGACACATATTAACACACACACTCCCCTCACAAGTATAAACAATAGTGGACCAAA
ATAAATTTTAACAACATACCCTGCATATTCTCACAACGTGGGGTCCGGGAAGGTAATGTGTACGCAGATC
TTACCTTTACCTTGTGAAGGTAGAGAAGTTATTTTTGATGGACTAACGGCTCAAGAAAAGCAGGACCAAA
GTCAAAGTCATTCAAAATTTTCTTCTATTTAATTTTGTTTTTTTCGCCTATATAGTTTTACATAGCAAAA
TCAGTCTCAAGCTCGAATAAAGAAGGGTTGCTATAAGCCGAAGTCAACTATGATGAATCCTTTGAATATT
GAGTTCACATAATGAATATAGAAAATCCATATATTTGAACCCCAAGTTATATGGAATTGACAAGCAATTA
ATTCTTCCCTCTGTCCAAGAACCAAACAAGTGAGGCAAAGAATTTACAAGTTCTTAATTATTCCTATTTT
TTGCTCCATTTAAATGGAAAATAATCAAGCATTTCCTCTAAGTCAACGAATTAATTAAGACGTGTGATGC
TAATAAGTCTAACTATCCCATACCCTCAACTTGCAATATCTTAGTTGGAGCAAATTNGGTCATACAACCC
ATACTATAATAGCATATAGCTTGGATATAGATGAAATGCTTTTTATAGAAGTCTTTACATTGAATGTGGG
GTCTCATTAAAATATATATAAGTTCAAAGAGAGTTAAACAAATGTATTTGTAATATCTAATGTGTAGATA
CATTAATTAAAGAATATATGTACATCAAAACAAAGGAGAGAAGACCACAGACTTTAATCATATGAAAAGG
ACAATCAAGACATCATTTTCAAATCCTAATTACATGCCACCCCCTTATGGCCAACTTCCCCCCCTATTTA
AACCTCTCTATTCCTCTAAACCCTCTCCACACATTTGTTTGTCAAACAACCAACTAGAGAACTTATATTC
CCTCTTGATCTCTTTTCTCCTCTTTGCTCTTTTGTCCCACTAAATCTATCCAAAAACAAAAATAGTACAT
CAAAAAAGTGAGTTATTTGCATATTTCAAGAACTTCTTTAATG
